# Risks and Benefits of Nalmefene in the Treatment of Adult Alcohol Dependence: A Systematic Literature Review and Meta-Analysis of Published and Unpublished Double-Blind Randomized Controlled Trials

**DOI:** 10.1371/journal.pmed.1001924

**Published:** 2015-12-22

**Authors:** Clément Palpacuer, Bruno Laviolle, Rémy Boussageon, Jean Michel Reymann, Eric Bellissant, Florian Naudet

**Affiliations:** 1 INSERM Centre d’Investigation Clinique 1414, Centre Hospitalier Universitaire de Rennes, Rennes, France; 2 Laboratoire de Pharmacologie Expérimentale et Clinique, Faculté de Médecine, Université de Rennes 1, Rennes, France; 3 Département de Médecine Générale, Faculté de Médecine et de Pharmacie, Université de Poitiers, Poitiers, France; University of Queensland, AUSTRALIA

## Abstract

**Background:**

Nalmefene is a recent option in alcohol dependence treatment. Its approval was controversial. We conducted a systematic review and meta-analysis of the aggregated data (registered as PROSPERO 2014:CRD42014014853) to compare the harm/benefit of nalmefene versus placebo or active comparator in this indication.

**Methods and Findings:**

Three reviewers searched for published and unpublished studies in Medline, the Cochrane Library, Embase, ClinicalTrials.gov, Current Controlled Trials, and bibliographies and by mailing pharmaceutical companies, the European Medicines Agency (EMA), and the US Food and Drug Administration. Double-blind randomized clinical trials evaluating nalmefene to treat adult alcohol dependence, irrespective of the comparator, were included if they reported (1) health outcomes (mortality, accidents/injuries, quality of life, somatic complications), (2) alcohol consumption outcomes, (3) biological outcomes, or (4) treatment safety outcomes, at 6 mo and/or 1 y. Three authors independently screened the titles and abstracts of the trials identified. Relevant trials were evaluated in full text. The reviewers independently assessed the included trials for methodological quality using the Cochrane Collaboration tool for assessing risk of bias. On the basis of the I2 index or the Cochrane’s Q test, fixed or random effect models were used to estimate risk ratios (RRs), mean differences (MDs), or standardized mean differences (SMDs) with 95% CIs. In sensitivity analyses, outcomes for participants who were lost to follow-up were included using baseline observation carried forward (BOCF); for binary measures, patients lost to follow-up were considered equal to failures (i.e., non-assessed patients were recorded as not having responded in both groups). Five randomized controlled trials (RCTs) versus placebo, with a total of 2,567 randomized participants, were included in the main analysis. None of these studies was performed in the specific population defined by the EMA approval of nalmefene, i.e., adults with alcohol dependence who consume more than 60 g of alcohol per day (for men) or more than 40 g per day (for women). No RCT compared nalmefene with another medication. Mortality at 6 mo (RR = 0.39, 95% CI [0.08; 2.01]) and 1 y (RR = 0.98, 95% CI [0.04; 23.95]) and quality of life at 6 mo (SF-36 physical component summary score: MD = 0.85, 95% CI [−0.32; 2.01]; SF-36 mental component summary score: MD = 1.01, 95% CI [−1.33; 3.34]) were not different across groups. Other health outcomes were not reported. Differences were encountered for alcohol consumption outcomes such as monthly number of heavy drinking days at 6 mo (MD = −1.65, 95% CI [−2.41; −0.89]) and at 1 y (MD = −1.60, 95% CI [−2.85; −0.35]) and total alcohol consumption at 6 mo (SMD = −0.20, 95% CI [−0.30; −0.10]). An attrition bias could not be excluded, with more withdrawals for nalmefene than for placebo, including more withdrawals for safety reasons at both 6 mo (RR = 3.65, 95% CI [2.02; 6.63]) and 1 y (RR = 7.01, 95% CI [1.72; 28.63]). Sensitivity analyses showed no differences for alcohol consumption outcomes between nalmefene and placebo, but the weight of these results should not be overestimated, as the BOCF approach to managing withdrawals was used.

**Conclusions:**

The value of nalmefene for treatment of alcohol addiction is not established. At best, nalmefene has limited efficacy in reducing alcohol consumption.

## Abstract 2

In a systematic review and meta-analysis, Florian Naudet and colleagues assess whether medication with the opioid antagonist nalmefene can reduce consumption and other outcomes of alcohol addiction.

## Editors' Summary

**Background:**

Many people enjoy an occasional alcoholic drink. But because alcohol is an addictive substance, some people (around one in 12 people in the US, for example) develop alcohol dependency (alcoholism). Such people have an excessive desire to drink or have lost control over their alcohol use, and may find it hard to relax or enjoy themselves without having a drink. As well as becoming psychologically dependent on alcohol, they can become physically dependent and may show withdrawal symptoms such as sweating, shaking, and nausea—or even delirium tremens, a psychotic condition that involves tremors, hallucinations, anxiety, and disorientation—when they attempt to reduce their drinking. Indeed, severely dependent drinkers often drink to relieve their withdrawal symptoms (“relief drinking”). Although alcohol dependency sometimes runs in families, it can also be triggered by stressful events, and the condition can damage health, emotional stability, finances, careers, and relationships.

**Why Was This Study Done?:**

To reduce harm, alcohol-dependent individuals are usually advised to abstain from drinking, but controlled (moderate) drinking may also be helpful. To help people reduce their alcohol consumption, the European Medicines Agency recently approved nalmefene—a drug that blocks the body’s opioid receptors and reduces the craving for alcohol—for use in the treatment of alcohol dependence in adults who consume more than 60 g (for men) or 40 g (for women) of alcohol per day (a small glass of wine contains about 12 g of alcohol; a can of beer contains about 16 g). However, several expert bodies have concluded that nalmefene shows no benefit over naltrexone, an older treatment for alcohol dependency, and do not recommend its use for this indication. Here, the researchers investigate the risks and benefits of nalmefene in the treatment of alcohol dependency in adults by undertaking a systematic review and meta-analysis of double-blind randomized controlled trials (RCTs) of nalmefene for this indication. A systematic review uses predefined criteria to identify all the research on a given topic, and a meta-analysis combines the results of several studies; a double-blind RCT compares outcomes in people chosen at random to receive different treatments without the researchers or the participants knowing who received which treatment until the end of the trial.

**What Did the Researchers Do and Find?:**

The researchers identified five RCTs that met the criteria for inclusion in their study. All five RCTs (which involved 2,567 participants) compared the effects of nalmefene with a placebo (dummy drug); none was undertaken in the population specified by the European Medicines Agency approval. Among the health outcomes examined in the meta-analysis, there were no differences between participants taking nalmefene and those taking placebo in mortality (death) after six months or one year of treatment, in the quality of life at six months, or in a summary score indicating mental health at six months. The RCTs included in the meta-analysis did not report other health outcomes such as accidents. Participants taking nalmefene had fewer heavy drinking days per month at six months and one year of treatment than participants taking placebo, and their total alcohol consumption was lower. However, more people withdrew from the nalmefene groups than from the placebo groups, often for safety reasons. Thus, attrition bias—selection bias caused by systematic differences between groups in withdrawals from a study that can affect the accuracy of the study’s findings—cannot be excluded. Indeed, when the researchers undertook an analysis in which they allowed for withdrawals, the alcohol consumption outcomes did not differ between the treatment groups.

**What Do These Findings Mean?:**

These findings show that there is no high-grade evidence currently available from RCTs to support the use of nalmefene for harm reduction among people being treated for alcohol dependency. In addition, they provide little evidence to support the use of nalmefene to reduce alcohol consumption among this population. Thus, the value of nalmefene for the treatment of alcohol addiction is not established. Importantly, these findings reveal a lack of information on clinically relevant outcomes in the evidence that led to nalmefene approval by the European Medicines Agency. Thus, these findings also call into question the decisions of this and other regulatory and advisory bodies that have approved nalmefene on the basis of the available evidence from RCTs, and highlight the need for further RCTs of nalmefene compared to placebo and naltrexone for the indication specified in the market approval.

**Additional Information:**

This list of resources contains links that can be accessed when viewing the PDF on a device or via the online version of the article at http://dx.doi.org/10.1371/journal.pmed.1001924.

- The US National Institute on Alcohol Abuse and Alcoholism has information about alcohol and its effects on health (including alcohol use disorder, another name for alcohol dependency); it provides interactive worksheets to help people evaluate their drinking and decide whether and how to make a change
- The UK National Health Service Choices website provides detailed information about drinking and alcohol and about alcohol dependency (including a personal story about alcohol misuse), and tools for calculating alcohol consumption
- The US National Council on Alcoholism and Drug Dependence provides information about alcohol addiction and a self-test for alcohol dependence
- Drinkaware is a UK-based non-profit organization that aims to improve the UK’s drinking habits; its website provides information on alcohol dependence and on other aspects of alcohol and health, and a tool for calculating alcohol intake
- MedlinePlus provides links to many other resources on alcohol and on alcoholism and alcohol abuse
- Wikipedia has pages on nalmefene and on naltrexone (note that Wikipedia is a free online encyclopedia that anyone can edit; available in several languages)
- Details of the European Medicine Agency approval of nalmefene are available
- More information about this study is available

## Introduction

The net effect of alcohol consumption on health is detrimental in terms of global mortality and global disability [1]. Harm reduction by controlled drinking is a concept that has been developed as an alternative to abstinence. While this approach still raises questions [2,3], nalmefene, an opioid antagonist, was recently approved by the European Medicines Agency (EMA) [4] for the treatment of alcohol dependence in order to help reduce alcohol consumption in adults who consume more than 60 g of alcohol per day (for men) or more than 40 g per day (for women). The UK National Institute for Health and Care Excellence (NICE) initially recommended it as a “possible” treatment for alcohol dependence [5], but, subsequently, the NICE evidence review group distanced itself from its earlier advice [6]. The French National Authority for Health Transparency Committee was also cautious and considered that nalmefene provided a minor improvement in actual benefit (ranking IV on a scale from I to V, with V = no improvement) compared to psychosocial support alone in the treatment of alcohol dependence. The Transparency Committee recommended that prescribing authority be restricted to addiction and alcohol specialists during the first year [7] (a recommendation that was not subsequently followed by the French Ministry of Social Affairs and Health). In contrast, the German Institute for Quality and Efficiency in Health Care concluded that studies of nalmefene showed no additional benefits for alcohol dependence compared to naltrexone, another opioid antagonist that was an earlier and less costly comparator therapy [8]. Furthermore, the Swedish Dental and Pharmaceutical Benefits Agency concluded that the lack of advantage of nalmefene compared to existing treatments suggested that it did not warrant recommendation for reimbursement [9]. Researchers’ opinions are more divided, with some arguing that nalmefene is a “paradigm shift” [10,11] and others claiming that it is a perfect example of “bad medicine” [12,13].

This state of affairs raises a fundamental problem. While phase III clinical testing should lead to objective conclusions concerning treatment effect, in the case of nalmefene, it has led to divergent and, indeed, antagonistic opinions. There are at present doubts about the clinical significance of differences in alcohol consumption outcomes reported in the different studies. In addition, nalmefene studies have not directly explored morbidity/mortality outcomes. These outcomes were recently proposed as relevant in the clinical evaluation of alcohol dependence treatments [14] and are indeed expected benefits of a harm reduction approach.

We therefore planned a meta-analysis of aggregated data to enable an objective reappraisal of the efficacy of nalmefene for relevant health outcomes, not solely restricted to alcohol consumption endpoints.

## Methods

We developed and followed a standard meta-analysis protocol (systematic review registration—PROSPERO 2014:CRD42014014853).

### Eligibility Criteria

#### Types of participants

We reviewed studies involving adults (aged 18 y and over) with a diagnosis of non-abstinent alcohol dependence.

#### Types of interventions

Studies were eligible if they focused on the comparison of oral nalmefene with either placebo or another active comparator. For multiple dose studies, only the dose closest to 20 mg per day (recommended dose in the EMA authorization) was considered. The EMA recommendation was based on data from dose–response studies that suggested that doses below this dosage were less efficacious [4]. However, no clear dose–response relationship could be established in terms of the endpoint monthly number of heavy drinking days (HDDs). We initially planned to include studies lasting 6 mo (±1 mo) and/or 1 y (±1 mo), as in the pivotal studies, because the expected benefits of nalmefene on health outcomes, and indeed in terms of public health, were not expected over shorter durations. Nonetheless, it appeared during the review process that there were a substantial number of trials over shorter periods. These studies were included in a post hoc analysis.

#### Types of outcomes

We considered four health outcomes as primary outcomes: (1) mortality, (2) accidents (including motor vehicle crashes) or injuries, (3) quality of life or functioning, and (4) somatic complications of alcoholism. These health outcomes were used earlier in a systematic review [14] and describe the expected clinical benefits of treatment of alcohol dependence.

Secondary outcomes were in three categories: alcohol consumption outcomes, biological outcomes, and treatment safety outcomes. The alcohol consumption outcomes were (1) monthly number of HDDs, defined as days with alcohol consumption of 60 g or more for males and 40 g or more for females, (2) total alcohol consumption, (3) response (responders are patients decreasing their consumption to low-risk levels or no consumption), (4) complete abstinence, (5) total Drinker Inventory of Consequences (DrInC) score, (6) Clinical Global Impression–Severity score, and (7) Alcohol Dependence Scale score. The biological outcomes were (1) γ-glutamyltransferase, (2) alanine-aminotransferase, (3) mean corpuscular volume, and (4) carbohydrate-deficient transferrin. The treatment safety outcomes were (1) treatment-emergent adverse events, (2) serious adverse events, (3) withdrawal from the study, and (4) withdrawal for safety reasons.

All outcomes were collected at both 6 mo (±1 mo) and 1 y (±1 mo). Studies reporting outcomes with follow-up of under 5 mo were considered only in a post hoc sensitivity analysis.

#### Types of studies

In this review, only randomized controlled trials (RCTs) were included. Studies were searched for without limitation in terms of date. Only study reports in English, French, or Spanish language were considered.

### Search Strategy

Eligible studies were identified from PubMed/Medline, the Cochrane Library and Embase, including conference abstracts. On PubMed, the keyword used was “nalmefene” with the filter “clinical trial.” Searches in other bases are detailed in S1 Supporting Information.

Unpublished studies were also searched for by communication with key organizations such as the US Food and Drug Administration and the “access to documents” service at the EMA, which granted us access to study reports of both published and unpublished clinical studies. The pharmaceutical companies Biotie Therapies and Lundbeck were contacted to provide information concerning their studies. A search on ClinicalTrials.gov and Current Controlled Trials was performed. When needed, the authors of abstracts were contacted for further information and were asked for the references of the studies. If no response was obtained to a first solicitation, they were contacted a second time.

### Study Selection

The eligibility assessment was performed independently in a blind standardized manner by two reviewers (C. P. and F. N.). Disagreements were resolved by consensus or in consultation with a third reviewer (B. L.). A comparison across studies, checking author names, treatment comparisons, sample sizes, and outcomes, was performed to avoid duplicates and compilations of data from several reports of the same study.

### Assessment of Methodological Quality

Each paper was then assessed for methodological quality prior to inclusion in the review using the Cochrane Collaboration tool for assessing risk of bias.

### Data Collection

A data extraction sheet based on the *Cochrane Handbook for Systematic Reviews of Interventions* guidelines [15] was used. Two review authors (C. P. and F. N.) extracted the data from the studies included. Disagreements were resolved by consensus or in consultation with a third reviewer (B. L.). For each study included, information was extracted on (1) characteristics of the study (year, country, type of comparator, number of arms, funding), (2) characteristics of trial participants (age, gender, number of patients included in the analysis, population analyzed), (3) type of intervention (treatment, duration), and (4) outcome measures as detailed above.

### Data Analysis

#### General strategy

The efficacy index used for binary outcomes was the risk ratio (RR), and the efficacy index used for continuous outcomes was the mean difference (MD), or standardized mean difference (SMD) when appropriate. These efficacy indices are reported with 95% confidence intervals. As a substantial number of patients lost to follow-up was expected, sensitivity analyses were planned using the baseline observation carried forward (BOCF) approach for continuous outcomes; for binary outcomes, loss to follow-up was considered as failure (i.e., non-assessed patients were recorded as not having responded in both groups).

#### Risk of bias across studies

We used visual inspection of the forest plots, the I2 index, and the Q statistic to investigate the possibility of statistical heterogeneity. In the absence of heterogeneity, we performed direct meta-analyses by synthesizing studies that compared the same interventions using a fixed effect model. When there was heterogeneity, we performed a direct meta-analysis with a random effect model.

Publication bias was not investigated graphically (funnel plots) nor by testing (the rank correlation test) because there was a small number of studies, which limits the usefulness of these methods [16].

Analyses were performed using R [17] with meta [18] and rmeta [19] libraries. Results are presented according to PRISMA (Preferred Reporting Items for Systematic Reviews and Meta-Analyses) format [20].

#### Minor changes to the initial protocol

During our searches, it appeared that some RCTs of nalmefene in the treatment of alcohol dependence also included patients with difficulty in controlling drinking, rather than alcohol dependence. These studies were considered if alcohol dependence was not an explicit exclusion criterion. For mortality, which is a rare outcome, a sensitivity analysis was performed using the gmeta library [21], which enables exact meta-analysis methods to be run. These methods can use all available information without artificial continuity correction on zero-event studies [22].

During the peer review process, the issue was raised of exploring a possible dose–response relationship between nalmefene and the outcomes. We therefore performed a networked meta-analysis on monthly number of HDDs, looking at different doses (particularly higher doses) and different administration regimens for nalmefene. This network meta-analysis was performed using the frequentist approach, which was implemented in R in the netmeta library [23].

## Results

### Study Selection

**Fig 1:**
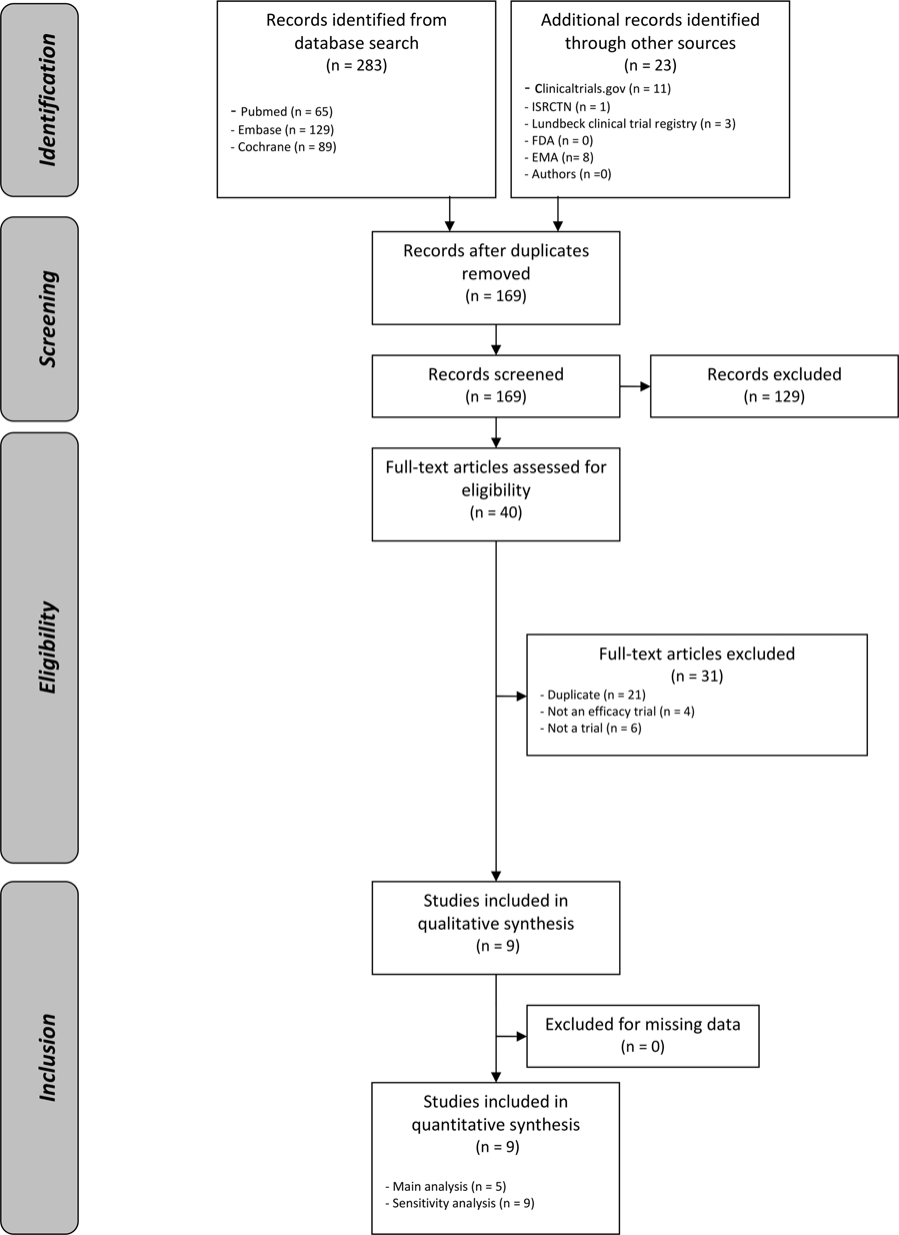
Flow diagram. FDA, US Food and Drug Administration.

After adjusting for duplicates, the searches provided a total of 169 citations. Of these, 129 studies were discarded (on the basis of title and abstract) because they did not meet the selection criteria. No study was discarded for language. After examination of the full text of the remaining 40 articles, 31 additional references were discarded. Nine studies were included in the quantitative review and five in the main analysis (studies with at least 6 mo of follow-up). Concerning unpublished data (missing outcomes in the publication or unpublished studies), clinical study reports were provided by the access to documents service at the EMA, and no supplemental information was retrieved by communication with study authors. A flowchart detailing the study selection process is given in Fig 1.

### Study Characteristics and Risk of Bias within Studies

The main analysis involved 2,567 randomized participants in five RCTs. All five studies provided data at 6 mo, and only one provided a 1-y follow-up. Four additional RCTs versus placebo (which did not meet the duration criterion) involving 353 additional randomized participants were also considered in the sensitivity analysis. All of these studies involved adults with alcohol dependence, but none was performed in the specific population defined by the EMA approval (i.e., adults with alcohol dependence consuming more than 60 g of alcohol per day for men or more than 40 g per day for women). In two of the studies included in our main analysis and one of the four studies added in the sensitivity analysis, patients were eligible based on alcohol dependence but also based on difficulty in controlling drinking, defined as (1) alcohol often taken in larger amounts or over a longer period than was intended or (2) a persistent desire or unsuccessful efforts to cut down or control drinking. No RCTs compared nalmefene with another active comparator.

The main characteristics of the different studies are presented in Table 1. The quality assessment of these studies is presented in Table 2. All of these studies were classified as presenting a high risk of incomplete outcome data because of large numbers of patients lost to follow-up.

**Table 1:** Summary of included studies evaluating the efficacy of nalmefene in the treatment of adult alcohol dependence (main and sensitivity analyses).

Study (EMA Study ID)	Participants	Nalmefene Dose*	Study Duration (Weeks)	Sponsor	Health Outcomes Reported in the Publication	Number of Patients	Age (Years), Mean ± Standard Deviation	Sex (Women), *n* (Percent)
Gual et al. [24] (12023A)^†^	“Patients were recruited from in- and out-patient clinics, from the study site’s patient pool, and by spontaneous referrals to the study site.”	20 mg/d (as needed)	24	Lundbeck	No	Nalmefene: 358; Placebo: 360	Nalmefene: 45.1 ± 10.7; Placebo: 44.4 ± 10.7	Nalmefene: 92 (26%); Placebo: 104 (29%)
Mann et al. [25] (12014A)^†^	“Patients were recruited from in- and out-patient clinics, including both spontaneous referrals and referrals resulting from special advertisements.”	20 mg/d (as needed)	24	Lundbeck	No	Nalmefene: 306; Placebo: 298	Nalmefene: 51.0 ± 10.1; Placebo: 52.1 ± 9.0	Nalmefene: 102 (33%); Placebo: 96 (32%)
Karhuvaara et al. [26] (CPH-101-801)^†^	“The subjects were recruited mainly by means of advertisements posted in newspapers.”	10 to 40 mg/d (as needed)	28	Biotie Therapies	No	Nalmefene: 242; Placebo: 161	Nalmefene: 49.5 ± 9.1; Placebo: 48.8 ± 8.4	Nalmefene: 46 (19%); Placebo: 29 (18%)
CPH-101-0701^†^	“Subjects were recruited through 3 channels: subjects were already patients of the investigator/referred to the sites by general practitioners/contacted the sites after seeing an advertisement.”	10 to 40 mg/d (as needed)	28	Biotie Therapies	Unpublished	Nalmefene: 85; Placebo: 82	Nalmefene: 45.8 ± 8.6; Placebo: 44.8 ± 10.4	Nalmefene: 33 (39%); Placebo: 31 (38%)
van den Brink et al. [27] (12013A)^†^	“Patients were recruited from outpatient clinics, from the study sites’ own patient pool, by referrals to the study site, or by using advertisements.”	20 mg/d (as needed)	52 + 4 wk of safety follow-up	Lundbeck	No	Nalmefene: 509; Placebo: 166	Nalmefene: 44.3 ± 11.2; Placebo: 44.3 ± 12	Nalmefene: 116 (23%); Placebo: 39 (23%)
Mason et al. [28]^‡^	“Potential subjects responding to Public Service Announcements advertising studies for alcoholics were evaluated for study inclusion and exclusion criteria.”	20 mg twice daily	12	Baker Norton Pharmaceuticals	No	Nalmefene: 7; Placebo: 7	42.0 ± 9.4**	6 (29%)**
Mason et al. [29]^‡^	“Subjects were alcohol-dependent outpatients recruited primarily through advertisements and press releases.”	10 mg or 40 mg twice daily	12	National grant and Ivax Corporation	No	Nalmefene: 70; Placebo: 35	Nalmefene: 41.9 ± 8.2; Placebo: 41.7 ± 9.9	Nalmefene: 22 (31%); Placebo: 13 (37%)
Anton et al. [30] (CPH-101-0299)^‡^	“Individuals were recruited from clinical referrals and direct advertisement.”	20 mg/d	12	Biotie Therapies	No	Nalmefene: 66; Placebo: 68	Nalmefene: 46.5 ± 10.9; Placebo: 45.1 ± 11.1	Nalmefene: 18 (27%); Placebo: 15 (22%)
CPH-101-0399^‡^	“Male and female subjects responding to advertisements were evaluated for their eligibility to participate.”	40 mg/d	16	Contral Pharma	Unpublished	Nalmefene: 50; Placebo: 50	Nalmefene: 50.0 ± 9.0; Placebo: 48.0 ± 9.0	Nalmefene: 6 (12%); Placebo: 11 (22%)

*Expressed in nalmefene HCL. Nalmefene HCL 20 mg corresponds to 18.06 mg nalmefene base.

^†^Studies included in the main and sensitivity analyses.

^‡^Studies included only in sensitivity analysis.

**Only overall data available.

**Table 2:** Quality assessment of included studies evaluating the efficacy of nalmefene in the treatment of adult alcohol dependence (main analysis).

Study	Sequence Generation	Allocation Concealment	Blinding	Incomplete Outcome Data	Selective Outcome Reporting	Other Source of Bias
Gual et al. (12023A)	Low risk	Low risk	Low risk	High risk	Low risk	Low risk
Mann et al. (12014A)	Low risk	Low risk	Low risk	High risk	Low risk	Low risk
Karhuvaara et al. (CPH-101-801)	Low risk	Low risk	Low risk	High risk	Low risk	Low risk
CPH-101-0701	Low risk	Low risk	Low risk	High risk	Low risk	Low risk
van den Brink et al. (12013A)	Low risk	Low risk	Low risk	High risk	Low risk	Low risk

Each study was assessed for methodological quality using a standardized critical appraisal instrument (the Cochrane Collaboration tool for assessing risk of bias).

### Results from Individual Studies and Synthesis of Results

#### Main analysis

**Fig 2:**
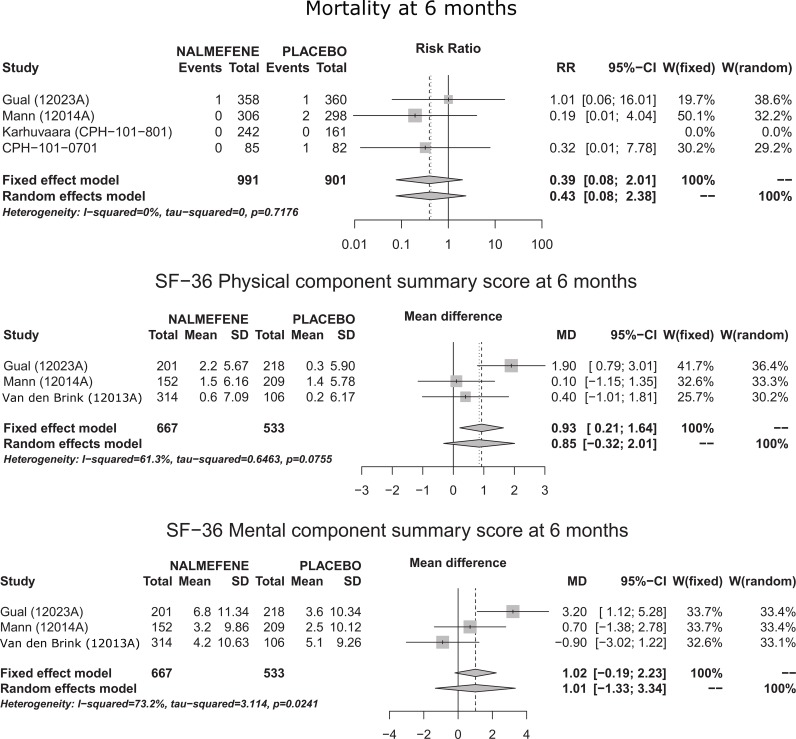
Forest plots for health outcomes at 6 mo.

Individual study results, direct meta-analyses (both fixed effect and random effect meta-analyses), and Q statistics are presented in detail in S1 Supporting Information for all outcomes. Concerning health outcomes (Fig 2), no difference was found across groups concerning mortality, and no results were extractable for accidents/injuries or for somatic alcoholism complications because these outcomes were not assessed. Quality of life was reported in three studies at 6 mo (but not at 1 y). Whereas one study showed superiority of nalmefene treatment over placebo on the SF-36 physical component summary score and the SF−36 mental component summary score, the other two studies did not replicate this finding—the summary measures for the two components did not provide evidence for any superiority (Fig 2; Table 3). Results concerning clinical, biological, and safety outcomes are reported in Table 3. As regards biological outcomes, only one study reported useable data concerning γ-glutamyltransferase, alanine-aminotransferase, and mean corpuscular volume.

**Table 3:** Summary of findings from trials assessing efficacy of nalmefene in the treatment of adult alcohol dependence.

Outcome	6-mo Follow-Up		1-y Follow-Up	
	Number of Studies	RR/MD/SMD (95% CI)	Number of Studies	RR/MD/SMD (95% CI)
**Health outcomes**				
Mortality (RR)	4	0.39 (0.08; 2.01) (F)	1	0.98 (0.04; 23.95) (F)
Accidents or injuries	—	Not available	—	Not available
QoL: SF−36 physical component summary score (MD)	3	0.85 (−0.32; 2.01) (R)	—	Not available
QoL: SF−36 mental component summary score (MD)	3	1.01 (−1.33; 3.34) (R)	—	Not available
Somatic alcoholism complications	—	Not available	—	Not available
**Alcohol consumption outcomes**				
Monthly number of HDDs (MD)	5	**−1.65 (−2.41; −0.89)** (F)	1	**−1.60 (−2.85; −0.35)** (F)
Total alcohol consumption (SMD)	5	**−0.20 (−0.30; −0.10)** (F)	1	−0.13 (−0.36; 0.1) (F)
Response (RR)	3	0.99 (0.88; 1.12) (R)	1	1.1 (0.99; 1.21) (F)
Complete abstinence	—	Not available	—	Not available
DrInC score (SMD)	5	**−0.12 (−0.22; −0.02)** (F)	—	Not available
Clinical Global Impression–Severity score (MD)	3	**−0.25 (−0.38; −0.13)** (F)	1	−0.20 (−0.44; 0.04) (F)
Alcohol Dependence Scale score (SMD)	4	**−0.11 (−0.21; −0.01)** (F)	—	Not available
**Biological outcomes**				
γ-Glutamyltransferase (MD)	1	**−38.2 (−75.74; −0.66)** (F)	—	Not available
Alanine-aminotransferase (MD)	1	**−14.5 (−23.69; −5.31)** (F)	—	Not available
Mean corpuscular volume (MD)	1	−0.20 (−1.29; 0.89) (F)	—	Not available
Carbohydrate-deficient transferrin (SMD)	4	−0.08 (−0.20; 0.05) (R)	1	−0.19 (−0.42; 0.05) (F)
**Treatment safety outcomes**				
Adverse events (RR)	4	**1.18 (1.11; 1.24)** (F)	1	**1.20 (1.05; 1.36)** (F)
Serious adverse events (RR)	4	0.89 (0.55; 1.43) (F)	1	1.39 (0.66; 2.94) (F)
Withdrawal from the study (RR)	4	**1.27 (1.01; 1.59)** (R)	1	1.20 (0.94; 1.53) (F)
Withdrawal from the study for safety reasons (RR)	4	**3.65 (2.02; 6.63)** (R)	1	**7.01 (1.72; 28.63)** (F)

All outcomes are presented at both 6 mo and 1 y as they were reported in the different studies. Efficiency indexes are presented with their 95 confidence intervals. Bold values are statistically significant.

F, fixed effect model; QoL, quality of life; R, random effect model.

#### Additional analyses

The sensitivity analyses (Table 4) using BOCF analysis were possible for a small number of efficacy outcomes (monthly number of HDDs, total alcohol consumption) and in only the three studies that reported these data. These analyses found no difference between nalmefene and placebo. Sensitivity analyses considering loss to follow-up as failure did not change the results concerning safety outcomes. Sensitivity analyses involving studies lasting less than 6 mo showed the robustness of our estimations, and are presented in S1 Supporting Information. For death, which was a rare outcome, we ran exact meta-analysis methods, which yielded the same results as the main analysis (i.e., no difference found between nalmefene and placebo).

**Table 4:** Summary of findings from sensitivity analyses using conservative approaches to managing withdrawals from the studies.

Outcome	RR/MD/SMD (95% CI)	
	6-mo Follow-Up	1-y Follow-Up
**Alcohol consumption outcomes**		
Monthly number of HDDs (MD)	−0.22 (−1.12; 0.68) (F, BOCF)	Not Available
Total alcohol consumption (SMD)	−0.01 (−0.11; 0.08) (F, BOCF)	Not Available
Response (RR)	0.92 (0.83; 1.02) (F, LFU = F)	Not Available
**Treatment safety outcomes**		
Adverse events (RR)	**1.17 (1.11; 1.23)** (F, LFU = F)	**1.20 (1.05; 1.36)** (F, LFU = F)
Serious adverse events (RR)	0.87 (0.60; 1.25) (F, LFU = F)	1.37 (0.7; 2.67) (F, LFU = F)

Bold values are statistically significant.

F, fixed effect model; LFU = F, lost to follow-up = failure.

#### Dose–response analysis

The main analysis was based on a set of very similar studies. Three of these used 20 mg nalmefene as needed, and two of them used adjustable dosages (20 mg, which could be adjusted to 10 mg or 40 mg if needed).

The network meta-analysis on monthly number of HDDs found no evidence for a dose–response relationship, although the possibility remains that the 10-mg daily dose could have been less efficacious than the 20 mg nalmefene as needed dosing. Network geometry and quantitative results are presented in detail in S2 Supporting Information.

## Discussion

### Summary of Evidence

Compared to placebo, there was no evidence for the efficacy of nalmefene on health outcomes. Nalmefene was shown to be slightly superior to placebo in reducing the monthly number of HDDs, total alcohol consumption, DrInC score, Clinical Global Impression–Severity score, and Alcohol Dependence Scale score. However, these findings were not robust and disappeared when a conservative approach to managing withdrawals was used.

Currently, there is a wide debate on whether abstinence or reduced drinking should be the aim of alcohol dependence treatment. Epidemiological studies have suggested that drinking-related outcomes were shown to determine unspecific health outcomes like quality of life in adolescents [31], but there is no high-quality randomized evidence concerning the efficacy of managed alcohol programs on their own on these health outcomes [32]. Without entering further into this debate [2], our results support the view that nalmefene RCTs give little evidence of a reduction in alcohol consumption and no evidence of “harm reduction” with nalmefene use. Additionally, no difference between nalmefene and placebo was found concerning serious adverse events. At best, evidence of “harm reduction” is provided by simulation studies [33,34].

In addition, we found no RCTs of nalmefene versus placebo conducted in the specific population defined by the EMA approval of nalmefene (i.e., adult patients with alcohol dependence who consume more than 60 g of alcohol per day for men or more than 40 g per day for women). In this population, the only available data are pooled subgroup analyses in two or three of the RCTs we identified [34,35]. However, the credibility of subgroup analyses, even when claims are strong, is usually low [36], and such analyses should be considered as exploratory rather than confirmatory. On the one hand, regarding efficacy, some studies have found lower efficacy of opioid antagonists in hazardous/harmful drinkers compared to more severely dependent drinkers [37,38], and this latter target population could be of interest. On the other hand, subgroup analyses raise important methodological issues, especially when they are not prespecified [39,40]. In the case of the nalmefene RCTs, the subgroup analyses were not defined a priori.

Finally, we found no RCTs comparing nalmefene with another medication as an active comparator. The current state of knowledge and treatment evidence suggest that there is little or no difference in efficacy in reducing heavy drinking between nalmefene and naltrexone [41]. Although naltrexone is not authorized for reducing drinking, as opposed to promoting abstinence, this is probably its main effect [42].

### Strengths and Limitations

Individual study results were very much influenced by the studies’ management of missing data. Most of the studies included presented a high rate of withdrawals. Whereas a loss to follow-up of 5% or lower is usually of little concern, a loss of 20% or more prevents good-quality intention-to-treat analysis, can cause biased estimates of the treatment effect [43], and restricts the scope for generalizing results [44]. There were more withdrawals, including more withdrawals for safety reasons, in the nalmefene group than in the placebo group in the included studies. In addition, extractable results were mainly derived from observed case analyses. These two characteristics expose studies to an attrition bias. Indeed, when patients with side effects are not included in the analysis, it can make a treatment appear to be effective when it is not. For this reason, we used a conservative approach to managing withdrawals based on a “worst” hypothesis. The BOCF approach could have nonetheless been overly conservative, as suggested recently [45], and its results, although informative, should be weighed accordingly.

Concerning our literature search, one might suspect a “Tower of Babel bias” [46] because only studies in English, French, and Spanish were considered. In fact, no study was excluded for this reason. Also, we did not use “Selincro,” the commercial name of nalmefene, as a keyword. In response to a reviewer’s comment before the publication of this paper, we conducted a post hoc search on PubMed with “nalmefene OR selincro,” and it provided the same number of references. All in all, the major strength of our analysis is that we included what we believe to be all available relevant data from completed studies of nalmefene meeting our inclusion criteria. Indeed, the EMA granted us permission to access all study reports. This also limits the risk of selective outcome reporting, despite the fact that in the identified publications, not all outcomes were reported.

It can be noted that we chose four primary outcomes (the four health outcomes) and that our analysis was not adjusted for multiple comparisons, with a possible inflation of type I error. We chose this approach to put equal emphasis on these health outcomes, which are the expected outcomes in terms of public health. While this is a limitation for our study, it also enables the issue of multiple comparisons to be tackled. This was problematic in the three pivotal studies on nalmefene for alcohol dependence [24,25,27], which used two co-primary outcomes without any specific adjustment for multiple comparisons.

In addition, none of the published reports presented all four health outcomes that were considered in this meta-analysis. In the published reports, data on mortality were accessible, but data concerning accidents/injuries, quality of life/functioning, and somatic alcoholism complications were not (or only partially) presented. One could argue that we could have extracted data on accidents/injuries and somatic alcoholism complications from serious adverse events described in the study reports, but post hoc reconstructions of the criteria in this way could have been biased. It can be said that this is a limitation of our meta-analysis, but it is rather one of the main results: the outcomes that are clinically relevant (i.e., outcomes that are not surrogate outcomes) are lacking in the primary evidence that led to the EMA’s nalmefene approval.

## Conclusion

**Implications for research.**This review calls into question the decisions of some of the regulatory and advisory bodies that have approved nalmefene on the basis of this evidence. Given our results, certain conditions should be set by health authorities for the maintenance of nalmefene market approval. In our opinion, RCTs against placebo and naltrexone proving the superiority of nalmefene in the approved indication are needed. In these studies, health outcomes should be assessed. These studies may be unrealistic from a methodological point of view because of the need for long-term follow-up and probably a large number of participants. There is thus a need for independent and well-designed post-marketing studies. In addition, a network meta-analysis is needed to assess the effectiveness of nalmefene in reducing alcohol consumption compared to the other active treatments available. We are currently performing this meta-analysis (see PROSPERO 2015:CRD42015019841 for more details).

**Clinical implications.** Clinicians must be aware that the value of nalmefene for the treatment of alcohol addiction is not established. At best, nalmefene has limited efficacy in reducing alcohol consumption.

## Supporting Information

S1 Supporting Information Supplementary text, tables, and figures. Includes further information regarding the search strategy and results from the main and sensitivity analyses.
(PDF)

S2 Supporting Information Dose–response analysis. (DOC)

S1 Text Study protocol. (PDF)

S2 Text PRISMA checklist. (DOC)

## Abbreviations

BOCF: baseline observation carried forward
DrInC: Drinker Inventory of Consequences
EMA: European Medicines Agency
HDD: heavy drinking day
MD: mean difference
NICE: National Institute for Health and Care Excellence
RCT: randomized controlled trial
RR: risk ratio
SMD: standardized mean difference
