# Supplementary material for: Risks and Benefits of Nalmefene in the Treatment of Adult Alcohol Dependence: A Systematic Literature Review and Meta-Analysis of Published and Unpublished Double-Blind Randomized Controlled Trials
Source: PLoS Med. 2015 Dec 22;12(12):e1001924. doi: 10.1371/journal.pmed.1001924 (PMC4687857; doi:10.1371/journal.pmed.1001924)

SEARCHES

Records identified from database search:

PubMed

Date: November 19, 2014

Keyword: nalmefene<br>Filters activated: Clinical Trial

Results: 65 references

EMBASE®

Date: November 21, 2014

KEY (nalmefen* OR 6-desoxy-6-methylenenaltrexone) AND (LIMIT-TO (EXACTKEYWORD , "Human") OR LIMIT-TO (EXACTKEYWORD , "Humans")) AND (LIMIT-TO (EXACTKEYWORD , "Clinical trial") OR LIMIT-TO (EXACTKEYWORD , "Controlled clinical trial") OR LIMIT-TO (EXACTKEYWORD , "Randomized controlled trial") OR LIMIT-TO (EXACTKEYWORD , "Major clinical study") OR LIMIT-TO (EXACTKEYWORD , "Clinical Trials") OR LIMIT-TO (EXACTKEYWORD , "Clinical article")) AND (EXCLUDE (EXACTKEYWORD , "Nonhuman") OR EXCLUDE (EXACTKEYWORD , "Animals")) AND (EXCLUDE (EXACTKEYWORD , "Review"))

Results: 129 references

THE COCHRANE LIBRARY
Independent high-quality evidence for health care decision making

Date: November 19, 2014

Keyword: nalmefene<br>Filters activated: Trials

Results: 89 references

ClinicalTrials.gov
A service of the U.S. National Institutes of Health

Date: November 27, 2014

Keyword: nalmefene

Results: 11 references
Retained: 3/8 references
NCT01969617 (COMPLETED) → EXCLUDED
NCT00811720 (ESENSE1)
NCT00812461 (ESENSE2)
NCT00811941 (SENSE)
NCT02197598 (RECRUITING) → EXCLUDED
NCT00000437 (COMPLETED) → EXCLUDED
NCT00000450 (COMPLETED) →EXCLUDED
NCT02195817 (RECRUITING) → EXCLUDED

ISRCTN registry

Date: November 27, 2014

Keyword: nalmefene

Results: 1 reference
Retained: 0 reference

Lundbeck Clinical Trial Registry

Date: November 27, 2014

Keyword: nalmefene

Results: 3 references
Retained: 3 references
Study ID: 12023A (ESENSE2)
Study ID: 12014A (ESENSE1)
Study ID: 12013A (SENSE)

Date: December 2, 2014
Sent here:
http://www.ema.europa.eu/ema/index.jsp?curl=pages/about_us/landing/ask_ema_landing_page.jsp&mid=WC0b01ac05806499f0
Request Reference Number: ASK-6656

Query:

Dear Sirs,

We are currently working on a meta-analysis to assess the efficacy of Nalmefene in the treatment of adults alcohol dependence in order to reduce alcohol consumption (Protocol Registration number on PROSPERO: CRD42014014853, http://www.crd.york.ac.uk/PROSPERO/display_record.asp?ID=CRD42014014853).

We carried out a literature search aimed at identifying the RCTs eligible for our analysis. This search was conducted in PubMed, Embase and the Cochrane Library. We also searched the ClinicalTrials.gov database and other relevant websites. Presently, we are attempting to identify further RCTs.

Would you be able to tell us if any clinical trial evaluating Nalmefene in the treatment of adults alcohol dependence was declared to your agency? If so, we would be very grateful if you could send us a copy of the results that are available. They will be carefully referenced.

We look forward to your answering and we thank you in advance.

We would like to express our gratitude for all your help in this matter,

Dr Florian Naudet, MD, PhD

Centre d'Investigation Clinique CIC-P INSERM 1414, Hôpital de Pontchaillou, Centre Hospitalier Universitaire de Rennes & Université de Rennes 1
2 Rue Henri le Guilloux 35000 Rennes
Rennes, France
Florian.NAUDET@chu-rennes.fr

Results: 8 references
Retained: 7 references

Date: December 2, 2014
Sent here:
Food and Drug Administration (FDA)
Sarah Kotler, Acting Director
Division of Freedom of Information, OES
U.S. Food & Drug Administration
5630 Fishers Lane
Room-1035
Rockville, Maryland 20857
301-796-3900 (main)
301-827-9267 (fax)

Query:

Food and Drug Administration (FDA)
Sarah Kotler, Acting Director
Division of Freedom of Information, OES
U.S. Food & Drug Administration
5630 Fishers Lane
Room-1035
Rockville, Maryland 20857
301-796-3900 (main)
301-827-9267 (fax)

Rennes, December 2014 the 2nd,

Dear Sirs,

We are currently working on a meta-analysis to assess the efficacy of Nalmefene in the treatment of adults alcohol dependence in order to reduce alcohol consumption (Protocol Registration number on PROSPERO: CRD42014014853, http://www.crd.york.ac.uk/PROSPERO/display_record.asp?ID=CRD42014014853).

We carried out a literature search aimed at identifying the RCTs eligible for our analysis. This search was conducted in PubMed, Embase and the Cochrane Library. We also searched the ClinicalTrials.gov database and other relevant websites. Presently, we are attempting to identify further RCTs.

Would you be able to tell us if any clinical trial evaluating Nalmefene in the treatment of adults alcohol dependence was declared to your agency? If so, we would be very grateful if you could send us a copy of the results that are available. They will be carefully referenced.

We look forward to your answering and we thank you in advance.

We would like to express our gratitude for all your help in this matter,

Dr Florian Naudet, MD, PhD

Service de psychiatrie adulte et de psychologie médicale
Centre d'Investigation Clinique CIC-P INSERM 1414, Hôpital de Pontchaillou, Centre Hospitalier Universitaire de Rennes & Université de Rennes 1
2 Rue Henri le Guilloux 35000 Rennes
Rennes, France
Florian.NAUDET@chu-rennes.fr

Results: 0 reference
Answer from FDA:

“My apologies. I had to be unexpectedly away from the office. Let me try and explain some of the issues with your FOIA request 2015-71. You are seeking information about whether there are any trials currently in process about a particular indication. FDA does not discuss information or release information about pending trials or applications. This type of information is generally considered to be confidential commercial information and therefore not releasable before a product is approved. I searched to see if we had any approved drug products for the treatment you have specified and I don’t see any for this product under the product name Nalfemene. There is a product Vivitrol (naltrexone) which was approved for a similar indication. If you would like the review package for that product we would be happy to send that to you under FOIA. Please confirm whether you would like the Vivitrol approval package.”

SUMMARY OF INCLUDED STUDIES

WebApp 1. Summary of included studies evaluating the efficacy of nalmefene in the treatment of adult alcohol dependence (main and sensitivity analyses).

Study (EMA Study ID)	Participants	Nalmefene Dose*	Study Duration (Weeks)	Sponsor	Health Outcomes Reported in the Publication	Number of Patients	Age (Years), Mean ± Standard Deviation	Sex (Women), *n* (Percent)
Gual et al. [24] (12023A)†	"Patients were recruited from in- and out-patient clinics, from the study site's patient pool, and by spontaneous referrals to the study site."	20 mg/d (as needed)	24	Lundbeck	No	Nalmefene: 358; placebo: 360	Nalmefene: 45.1 ± 10.7; placebo: 44.4 ± 10.7	Nalmefene: 92 (26%); placebo: 104 (29%)
Mann et al. [25] (12014A)†	"Patients were recruited from in- and out-patient clinics, including both spontaneous referrals and referrals resulting from special advertisements."	20 mg/d (as needed)	24	Lundbeck	No	Nalmefene: 306; placebo: 298	Nalmefene: 51.0 ± 10.1; placebo: 52.1 ± 9.0	Nalmefene: 102 (33%); placebo: 96 (32%)
Karhuvaara et al. [26] (CPH-101-801)†	"The subjects were recruited mainly by means of advertisements posted in newspapers."	10 to 40 mg/d (as needed)	28	Biotie Therapies	No	Nalmefene: 242; placebo: 161	Nalmefene: 49.5 ± 9.1; placebo: 48.8 ± 8.4	Nalmefene: 46 (19%); placebo: 29 (18%)
CPH-101-0701†	"Subjects were recruited through 3 channels: subjects were already patients of the investigator/referred to the sites by general practitioners/contacted the sites after seeing an advertisement."	10 to 40 mg/d (as needed)	28	Biotie Therapies	Unpublished	Nalmefene: 85; placebo: 82	Nalmefene: 45.8 ± 8.6; placebo: 44.8 ± 10.4	Nalmefene: 33 (39%); placebo: 31 (38%)
van den Brink et al. [27] (12013A)†	"Patients were recruited from outpatient clinics, from the study sites' own patient pool, by referrals to the study site, or by using advertisements."	20 mg/d (as needed)	52 + 4 wk of safety follow-up	Lundbeck	No	Nalmefene: 509; placebo: 166	Nalmefene: 44.3 ± 11.2; placebo: 44.3 ± 12	Nalmefene: 116 (23%); placebo: 39 (23%)
Mason et al. [28]‡	"Potential subjects responding to Public Service Announcements advertising studies for alcoholics were evaluated for study inclusion and exclusion criteria."	20 mg twice daily	12	Baker Norton Pharmaceuticals	No	Nalmefene: 7; placebo: 7	42.0 ± 9.4**	6 (29%)**
Mason et al. [29]‡	"Subjects were alcohol-dependent outpatients recruited primarily through advertisements and press releases."	10 mg or 40 mg twice daily	12	National grant and Ivax Corporation	No	Nalmefene: 70; placebo: 35	Nalmefene: 41.9 ± 8.2; placebo: 41.7 ± 9.9	Nalmefene: 22 (31%); placebo: 13 (37%)
Anton et al. [30] (CPH-101-0299)‡	"Individuals were recruited from clinical referrals and direct advertisement."	20 mg/d	12	Biotie Therapies	No	Nalmefene: 66; placebo: 68	Nalmefene: 46.5 ± 10.9; placebo: 45.1 ± 11.1	Nalmefene: 18 (27%); placebo: 15 (22%)
CPH-101-0399‡	"Male and female subjects responding to advertisements were evaluated for their eligibility to participate."	40 mg/d	16	Contral Pharma	Unpublished	Nalmefene: 50; placebo: 50	Nalmefene: 50.0 ± 9.0; placebo: 48.0 ± 9.0	Nalmefene: 6 (12%); placebo: 11 (22%)

*Expressed in nalmefene HCL. Nalmefene HCL 20 mg corresponds to 18.06 mg nalmefene base. **Only overall data available.

†Studies included in the main and sensitivity analyses. ‡Studies included only in sensitivity analysis.

WebApp 2. Quality assessment of included studies evaluating the efficacy of nalmefene in the treatment of adult alcohol dependence.

Study	Sequence generation	Allocation concealment	Blinding	Incomplete outcome data	Selective outcome reporting	Other source of bias
Gual et al. (12023A)	Low risk	Low risk	Low risk	High risk	Low risk	Low risk
Mann et al. (12014A)	Low risk	Low risk	Low risk	High risk	Low risk	Low risk
Karhuvaara et al. (CPH-101-801)	Low risk	Low risk	Low risk	High risk	Low risk	Low risk
CPH-101-0701	Low risk	Low risk	Low risk	High risk	Low risk	Low risk
van den Brink et al. (12013A)	Low risk	Low risk	Low risk	High risk	Low risk	Low risk
Mason et al. (1994)	Unclear risk	Unclear risk	Low risk	Unclear risk	High risk	Low risk
Mason et al. (1999)	Unclear risk	Unclear risk	Low risk	Unclear risk	High risk	Low risk
Anton et al. (CPH-101-0299)	Unclear risk	Low risk	Low risk	High risk	Low risk	Low risk
CPH-101-0399	Low risk	Low risk	Low risk	Low risk	Low risk	Low risk

Each study was assessed for methodological quality using the Cochrane Collaboration tool for assessing risk of bias.

MAIN ANALYSIS

Mortality at 6 months

Study	NALMEFENE Events	NALMEFENE Total	PLACEBO Events	PLACEBO Total	RR	95%−CI	W(fixed)	W(random)
Gual (12023A)	1	358	1	360	1.01	[0.06; 16.01]	19.7%	38.6%
Mann (12014A)	0	306	2	298	0.19	[0.01; 4.04]	50.1%	32.2%
Karhuvaara (CPH−101−801)	0	242	0	161			0.0%	0.0%
CPH−101−0701	0	85	1	82	0.32	[0.01; 7.78]	30.2%	29.2%
**Fixed effect model**		**991**		**901**	**0.39**	**[0.08; 2.01]**	**100%**	**−−**
**Random effects model**					**0.43**	**[0.08; 2.38]**	**−−**	**100%**

Heterogeneity: I−squared=0%, tau−squared=0, p=0.7176

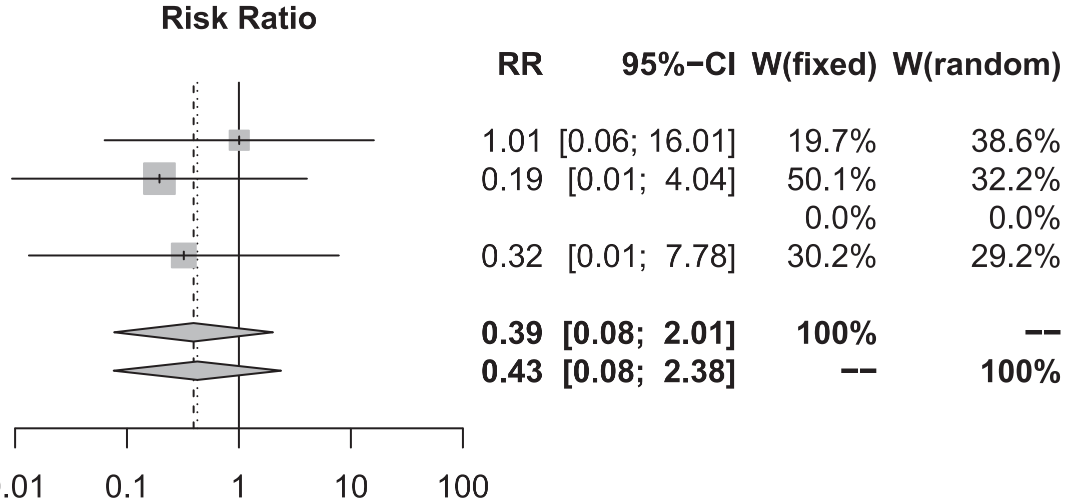

QoL:SF−36 Physical component summary score at 6 months

	NALMEFENE			PLACEBO						
**Study**	**Total**	**Mean**	**SD**	**Total**	**Mean**	**SD**	**MD**	**95%−CI**	**W(fixed)**	**W(random)**
Gual (12023A)	201	2.2	5.67	218	0.3	5.90	1.90	[0.79; 3.01]	41.7%	36.4%
Mann (12014A)	152	1.5	6.16	209	1.4	5.78	0.10	[−1.15; 1.35]	32.6%	33.3%
Van den Brink (12013A)	314	0.6	7.09	106	0.2	6.17	0.40	[−1.01; 1.81]	25.7%	30.2%
**Fixed effect model**	**667**			**533**			**0.93**	**[0.21; 1.64]**	**100%**	**--**
**Random effects model**							**0.85**	**[−0.32; 2.01]**	**--**	**100%**

Heterogeneity: I−squared=61.3%, tau−squared=0.6463, p=0.0755

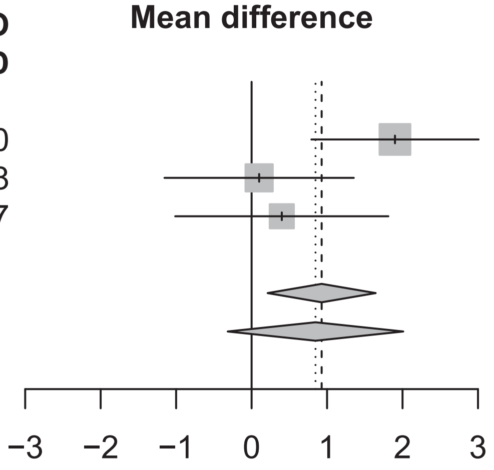

QoL:SF−36 Mental component summary score at 6 months

Study	NALMEFENE Total	NALMEFENE Mean	NALMEFENE SD	PLACEBO Total	PLACEBO Mean	PLACEBO SD	MD	95%−CI	W(fixed)	W(random)
Gual (12023A)	201	6.8	11.34	218	3.6	10.34	3.20	[1.12; 5.28]	33.7%	33.4%
Mann (12014A)	152	3.2	9.86	209	2.5	10.12	0.70	[−1.38; 2.78]	33.7%	33.4%
Van den Brink (12013A)	314	4.2	10.63	106	5.1	9.26	−0.90	[−3.02; 1.22]	32.6%	33.1%
**Fixed effect model**	**667**			**533**			**1.02**	**[−0.19; 2.23]**	**100%**	**−−**
**Random effects model**							**1.01**	**[−1.33; 3.34]**	**−−**	**100%**

Heterogeneity: I−squared=73.2%, tau−squared=3.114, p=0.0241

Mean difference

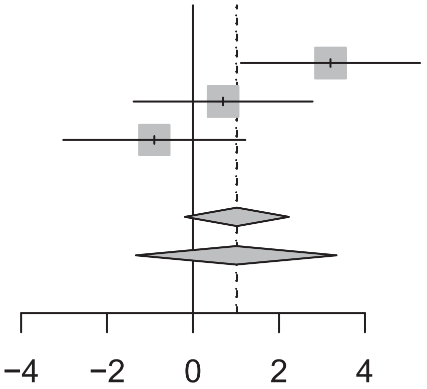

Monthly number of HDDs at 6 months

	NALMEFENE			PLACEBO						
**Study**	**Total**	**Mean**	**SD**	**Total**	**Mean**	**SD**	**MD**	**95%−CI**	**W(fixed)**	**W(random)**
Gual (12023A)	212	−12.3	7.3	229	−10.6	7.6	−1.70	[−3.09; −0.31]	30.1%	30.1%
Mann (12014A)	152	−11.2	7.4	213	−8.9	8.8	−2.30	[−3.97; −0.63]	20.9%	20.9%
Van den Brink (12013A)	320	−9.8	7.2	110	−8.9	6.3	−0.90	[−2.32; 0.52]	29.0%	29.0%
Karhuvaara (CPH−101−801)	147	−6.1	7.7	114	−4.2	7.4	−1.90	[−3.74; −0.06]	17.1%	17.1%
CPH−101−0701	30	−10.9	9.3	37	−8.5	9.1	−2.40	[−6.84; 2.04]	3.0%	3.0%
**Fixed effect model**	**861**			**703**			**−1.65**	**[−2.41; −0.89]**	**100%**	**−−**
**Random effects model**							**−1.65**	**[−2.41; −0.89]**	**−−**	**100%**

Heterogeneity: I−squared=0%, tau−squared=0, p=0.7642

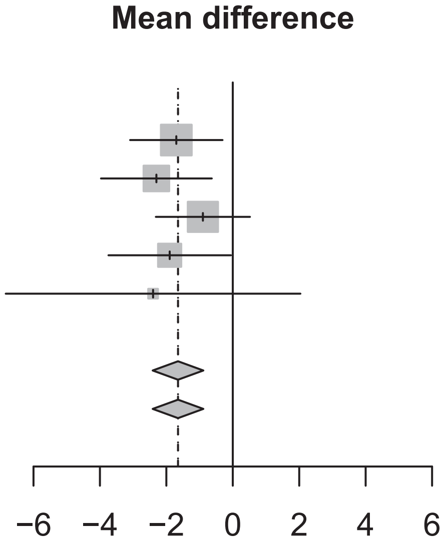

Monthly number of HDDs (BOCF) at 6 months

Study	NALMEFENE Total	NALMEFENE Mean	NALMEFENE SD	PLACEBO Total	PLACEBO Mean	PLACEBO SD	MD	95%−CI	W(fixed)	W(random)
Gual (12023A)	329	−8.3	10.9	326	−7.6	10.8	−0.70	[−2.36; 0.96]	29.5%	29.5%
Mann (12014A)	290	−6.1	10.2	289	−6.2	10.2	0.10	[−1.56; 1.76]	29.5%	29.5%
Van den Brink (12013A)	415	−8.0	8.1	137	−7.9	7.0	−0.10	[−1.51; 1.31]	41.1%	41.1%
**Fixed effect model**	**1034**			**752**			**−0.22**	**[−1.12; 0.68]**	**100%**	**−−**
**Random effects model**							**−0.22**	**[−1.12; 0.68]**	**−−**	**100%**

Heterogeneity: I−squared=0%, tau−squared=0, p=0.7824

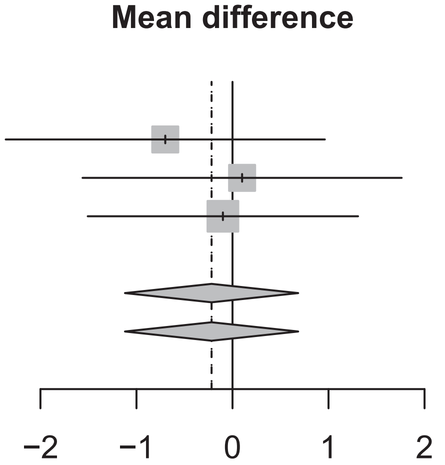

Total alcohol consumption at 6 months

Study	NALMEFENE Total	NALMEFENE Mean	NALMEFENE SD	PLACEBO Total	PLACEBO Mean	PLACEBO SD	SMD	95%−CI	W(fixed)	W(random)
Gual (12023A)	212	−59.0	33.5	229	−54.0	33.3	−0.15	[−0.34; 0.04]	30.5%	30.5%
Mann (12014A)	152	−50.7	29.6	213	−39.7	32.1	−0.35	[−0.56; −0.14]	24.3%	24.3%
Van den Brink (12013A)	320	−49.0	28.6	110	−45.6	27.3	−0.12	[−0.34; 0.10]	22.7%	22.7%
Karhuvaara (CPH−101−801)	149	−16.3	24.0	114	−11.3	22.5	−0.21	[−0.46; 0.03]	17.9%	17.9%
CPH−101−0701	30	−44.5	48.0	37	−41.6	49.0	−0.06	[−0.54; 0.42]	4.6%	4.6%
**Fixed effect model**	**863**			**703**			**−0.20**	**[−0.30; −0.10]**	**100%**	**−−**
**Random effects model**							**−0.20**	**[−0.30; −0.10]**	**−−**	**100%**

Heterogeneity: I−squared=0%, tau−squared=0, p=0.5261

Standardised mean difference

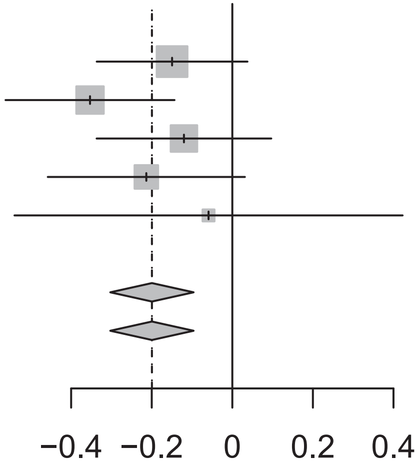

Total alcohol consumption (BOCF) at 6 months

Study	NALMEFENE Total	NALMEFENE Mean	NALMEFENE SD	PLACEBO Total	PLACEBO Mean	PLACEBO SD	SMD	95%−CI	W(fixed)	W(random)
Gual (12023A)	329	−40.2	50.8	326	−37.1	50.6	−0.06	[−0.21; 0.09]	39.8%	39.8%
Mann (12014A)	290	−26.1	42.6	289	−27.0	42.5	0.02	[−0.14; 0.18]	35.2%	35.2%
Van den Brink (12013A)	415	−37.9	42.8	137	−38.3	37.5	0.01	[−0.18; 0.20]	25.0%	25.0%
**Fixed effect model**	**1034**			**752**			**−0.01**	**[−0.11; 0.08]**	**100%**	**−−**
**Random effects model**							**−0.01**	**[−0.11; 0.08]**	**−−**	**100%**

Heterogeneity: I−squared=0%, tau−squared=0, p=0.7413

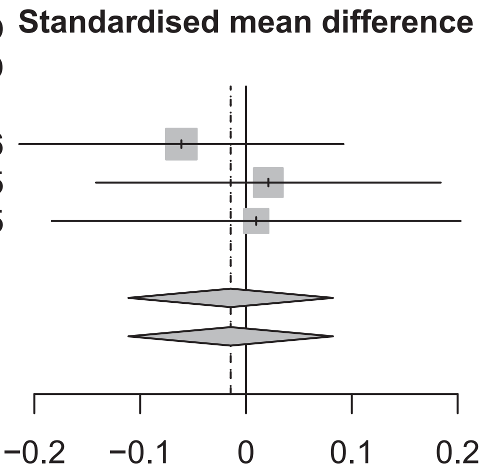

Response at 6 months

Study	NALMEFENE Events	NALMEFENE Total	PLACEBO Events	PLACEBO Total	RR	95%−CI	W(fixed)	W(random)
Gual (12023A)	221	329	206	326	1.06	[0.95; 1.19]	42.1%	38.3%
Mann (12014A)	107	290	128	289	0.83	[0.68; 1.02]	26.1%	22.3%
Van den Brink (12013A)	324	415	104	137	1.03	[0.92; 1.14]	31.8%	39.4%
**Fixed effect model**		**1034**		**752**	**0.99**	**[0.92; 1.07]**	**100%**	**−−**
**Random effects model**					**0.99**	**[0.88; 1.12]**	**−−**	**100%**

Heterogeneity: I−squared=58.9%, tau−squared=0.0064, p=0.0876

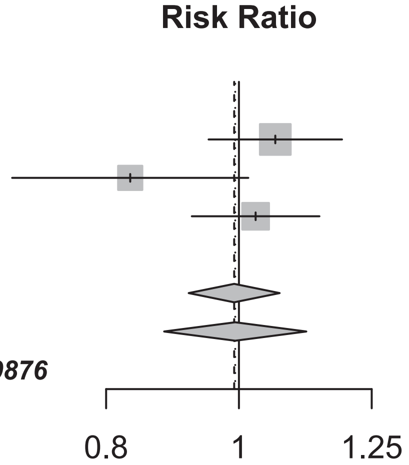

Response (LFU=F) at 6 months

Study	NALMEFENE Events	NALMEFENE Total	PLACEBO Events	PLACEBO Total	RR	95%−CI	W(fixed)	W(random)
Gual (12023A)	150	358	156	360	0.97	[0.82; 1.15]	37.4%	36.4%
Mann (12014A)	107	306	128	298	0.81	[0.67; 1.00]	31.1%	26.5%
Van den Brink (12013A)	259	509	87	166	0.97	[0.82; 1.15]	31.5%	37.1%
**Fixed effect model**		**1173**		**824**	**0.92**	**[0.83; 1.02]**	**100%**	**−−**
**Random effects model**					**0.93**	**[0.83; 1.03]**	**−−**	**100%**

Heterogeneity: I−squared=6.6%, tau−squared=0.0006, p=0.3429

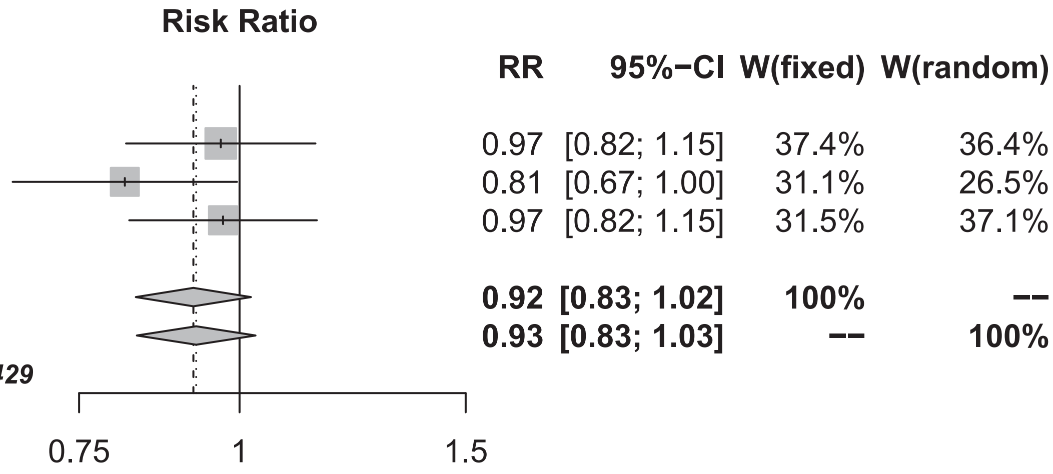

DrInC score at 6 months

Study	NALMEFENE Total	NALMEFENE Mean	NALMEFENE SD	PLACEBO Total	PLACEBO Mean	PLACEBO SD	SMD	95%−CI	W(fixed)	W(random)
Gual (12023A)	209	−20.4	21.7	230	−19.0	22.7	−0.06	[−0.25; 0.12]	29.5%	27.8%
Mann (12014A)	158	−16.0	13.8	213	−13.8	14.6	−0.15	[−0.36; 0.05]	24.4%	24.0%
Van den Brink (12013A)	318	−18.8	23.2	108	−19.5	21.8	0.03	[−0.19; 0.25]	21.7%	21.9%
Karhuvaara (CPH−101−801)	161	24.7	17.4	115	30.2	20.1	−0.30	[−0.54; −0.05]	17.9%	18.7%
CPH−101−0701	49	29.5	23.5	47	36.5	28.0	−0.27	[−0.67; 0.13]	6.4%	7.5%
**Fixed effect model**	**895**			**713**			**−0.12**	**[−0.22; −0.02]**	**100%**	**--**
**Random effects model**							**−0.12**	**[−0.24; −0.01]**	**--**	**100%**

Heterogeneity: I−squared=17.7%, tau−squared=0.003, p=0.3022

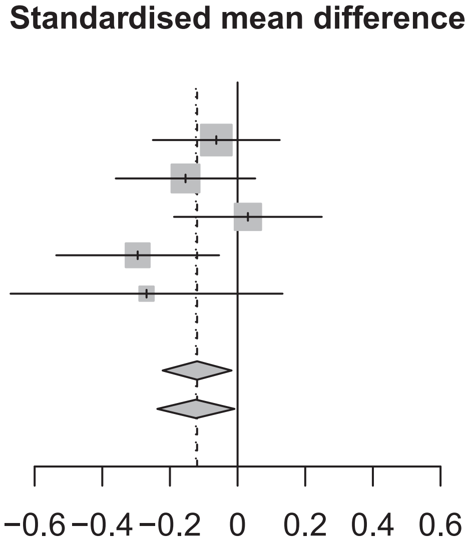

Clinical Global Impression−Severity score at 6 months

	NALMEFENE			PLACEBO						
**Study**	**Total**	**Mean**	**SD**	**Total**	**Mean**	**SD**	**MD**	**95%−CI**	**W(fixed)**	**W(random)**
Gual (12023A)	203	−1.27	1.4	225	−1.04	1.5	−0.23	[−0.50; 0.04]	21.3%	21.3%
Mann (12014A)	152	−1.27	1.0	210	−0.90	1.2	−0.37	[−0.60; −0.14]	31.2%	31.2%
Van den Brink (12013A)	306	−0.94	0.9	104	−0.75	0.8	−0.19	[−0.37; −0.01]	47.6%	47.6%
**Fixed effect model**	**661**			**539**			**−0.25**	**[−0.38; −0.13]**	**100%**	**−−**
**Random effects model**							**−0.25**	**[−0.38; −0.13]**	**−−**	**100%**

Heterogeneity: I−squared=0%, tau−squared=0, p=0.4733

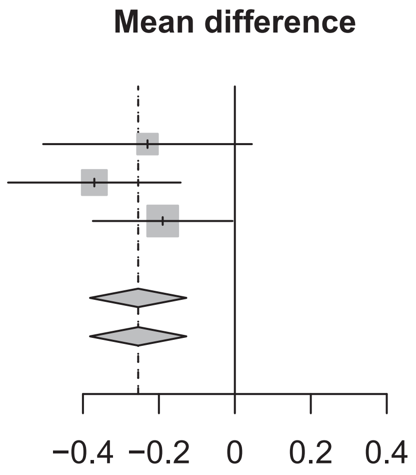

Alcohol Dependence Scale score at 6 months

Study	NALMEFENE Total	NALMEFENE Mean	NALMEFENE SD	PLACEBO Total	PLACEBO Mean	PLACEBO SD	SMD	95%−CI	W(fixed)	W(random)
Gual (12023A)	328	−5.9	7.2	325	−5.1	7.2	−0.11	[−0.26; 0.04]	40.6%	40.6%
Mann (12014A)	290	−4.6	5.1	289	−4.3	5.1	−0.06	[−0.22; 0.10]	36.1%	36.1%
Karhuvaara (CPH−101−801)	167	11.6	6.7	120	13.1	7.0	−0.22	[−0.45; 0.02]	17.3%	17.3%
CPH−101−0701	49	12.2	9.0	47	13.3	9.5	−0.12	[−0.52; 0.28]	6.0%	6.0%
**Fixed effect model**	**834**			**781**			**−0.11**	**[−0.21; −0.01]**	**100%**	**−−**
**Random effects model**							**−0.11**	**[−0.21; −0.01]**	**−−**	**100%**

Heterogeneity: I−squared=0%, tau−squared=0, p=0.7511

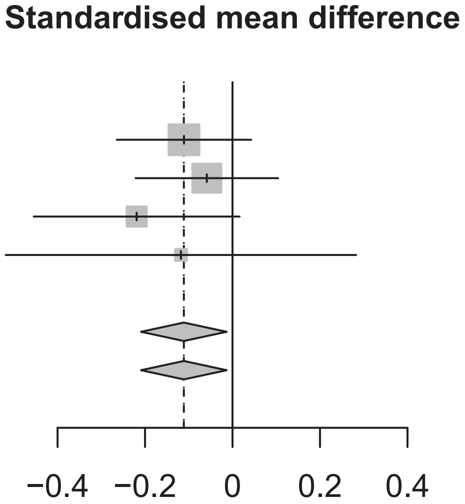

Gamma−Glutamyltransferase at 6 months

Study	NALMEFENE Total	NALMEFENE Mean	NALMEFENE SD	PLACEBO Total	PLACEBO Mean	PLACEBO SD	MD	95%−CI	W(fixed)	W(random)
Gual (12023A)	207	43.4	NA	224	44.9	NA	−1.5		0%	0%
Mann (12014A)	158	40.3	NA	211	45.7	NA	−5.4		0%	0%
Van den Brink (12013A)	319	32.2	NA	108	34.5	NA	−2.3		0%	0%
Karhuvaara (CPH−101−801)	148	77.2	89.1	115	115.4	189.8	−38.2	[−75.74; −0.66]	100%	100%
**Fixed effect model**	**832**			**658**			**−38.2**	**[−75.74; −0.66]**	**100%**	**−−**
**Random effects model**							**−38.2**	**[−75.74; −0.66]**	**−−**	**100%**

Heterogeneity: I−squared=NaN%, tau−squared=0, p=1

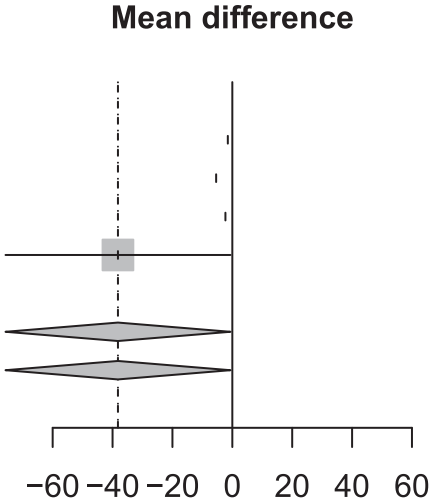

Alanine−aminotransferase at 6 months

Study	NALMEFENE Total	NALMEFENE Mean	NALMEFENE SD	PLACEBO Total	PLACEBO Mean	PLACEBO SD	MD	95%−CI	W(fixed)	W(random)
Gual (12023A)	205	25.0	NA	222	27.2	NA	−2.2		0%	0%
Mann (12014A)	158	25.4	NA	209	28.1	NA	−2.7		0%	0%
Van den Brink (12013A)	318	25.6	NA	108	25.8	NA	−0.2		0%	0%
Karhuvaara (CPH−101−801)	148	37.5	24.5	115	52.0	45.4	−14.5	[−23.69; −5.31]	100%	100%
**Fixed effect model**	**829**			**654**			**−14.5**	**[−23.69; −5.31]**	**100%**	**−−**
**Random effects model**							**−14.5**	**[−23.69; −5.31]**	**−−**	**100%**

Heterogeneity: I−squared=NaN%, tau−squared=0, p=1

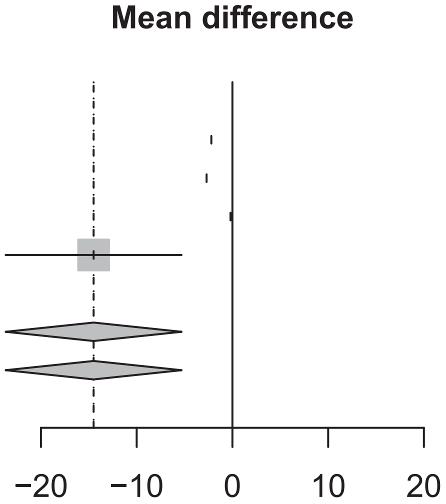

Mean corpuscular volume at 6 months

Study	NALMEFENE Total	NALMEFENE Mean	NALMEFENE SD	PLACEBO Total	PLACEBO Mean	PLACEBO SD	MD	95%−CI	W(fixed)	W(random)
Gual (12023A)	193	97.2	NA	215	97.3	NA	−0.1		0%	0%
Mann (12014A)	145	96.5	NA	204	96.7	NA	−0.2		0%	0%
Van den Brink (12013A)	296	95.5	NA	103	95.6	NA	−0.1		0%	0%
Karhuvaara (CPH−101−801)	147	96.1	3.8	114	96.3	4.9	−0.2	[−1.29; 0.89]	100%	100%
**Fixed effect model**	**781**			**636**			**−0.2**	**[−1.29; 0.89]**	**100%**	**--**
**Random effects model**							**−0.2**	**[−1.29; 0.89]**	**--**	**100%**

Heterogeneity: I−squared=NaN%, tau−squared=0, p=1

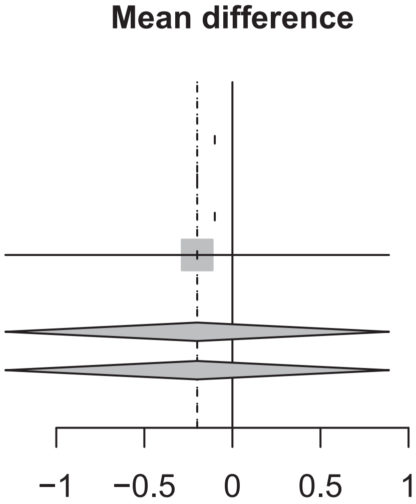

Carbohydrate−deficient transferrin at 6 months

**Study**	**NALMEFENE Total**	**NALMEFENE Mean**	**NALMEFENE SD**	**PLACEBO Total**	**PLACEBO Mean**	**PLACEBO SD**	**SMD**	**95%−CI**	**W(fixed)**	**W(random)**
Gual (12023A)	195	0.10	1.4	216	0.00	1.5	0.07	[−0.12; 0.26]	30.7%	29.1%
Mann (12014A)	152	0.00	1.2	207	0.10	1.4	−0.08	[−0.29; 0.13]	26.3%	26.1%
Van den Brink (12013A)	313	−0.14	1.2	106	0.13	1.1	−0.23	[−0.45; −0.01]	23.6%	24.1%
Karhuvaara (CPH−101−801)	148	3.10	1.8	115	3.30	2.0	−0.11	[−0.35; 0.14]	19.4%	20.7%
**Fixed effect model**	**808**			**644**			**−0.07**	**[−0.18; 0.03]**	**100%**	**--**
**Random effects model**							**−0.08**	**[−0.20; 0.05]**	**--**	**100%**

Heterogeneity: I−squared=25.9%, tau−squared=0.0042, p=0.2563

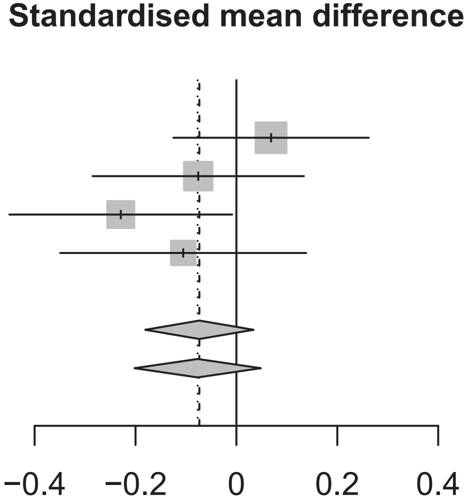

Adverse events at 6 months

Study	NALMEFENE Events	NALMEFENE Total	PLACEBO Events	PLACEBO Total	RR	95%−CI	W(fixed)	W(random)
Gual (12023A)	232	341	199	337	1.15	[1.03; 1.29]	32.2%	19.3%
Mann (12014A)	246	302	198	296	1.22	[1.11; 1.34]	32.2%	27.4%
Karhuvaara (CPH−101−801)	230	242	131	161	1.17	[1.08; 1.26]	25.3%	40.6%
CPH−101−0701	75	85	63	82	1.15	[1.00; 1.32]	10.3%	12.7%
**Fixed effect model**		**970**		**876**	**1.18**	**[1.11; 1.24]**	**100%**	**−−**
**Random effects model**					**1.18**	**[1.12; 1.24]**	**−−**	**100%**

Heterogeneity: I−squared=0%, tau−squared=0, p=0.859

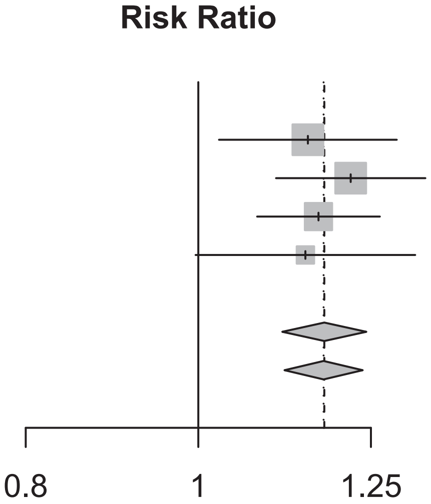

Adverse events (LFU=F) at 6 months

Study	NALMEFENE Events	NALMEFENE Total	PLACEBO Events	PLACEBO Total	RR	95%−CI	W(fixed)	W(random)
Gual (12023A)	249	358	222	360	1.13	[1.01; 1.25]	34.3%	21.7%
Mann (12014A)	250	306	200	298	1.22	[1.11; 1.34]	31.4%	27.0%
Karhuvaara (CPH−101−801)	230	242	131	161	1.17	[1.08; 1.26]	24.4%	39.1%
CPH−101−0701	75	85	63	82	1.15	[1.00; 1.32]	9.9%	12.2%
**Fixed effect model**		**991**		**901**	**1.17**	**[1.11; 1.23]**	**100%**	**−−**
**Random effects model**					**1.17**	**[1.11; 1.23]**	**−−**	**100%**

Heterogeneity: I−squared=0%, tau−squared=0, p=0.7556

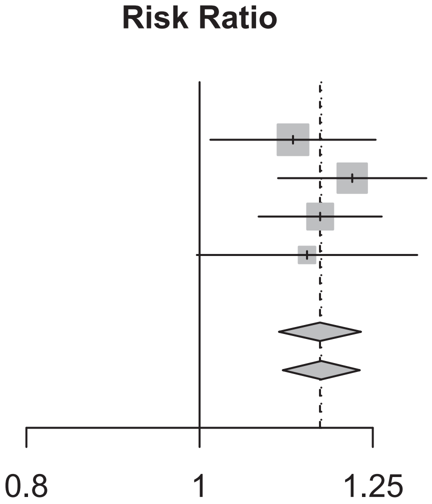

Serious adverse events at 6 months

Study	NALMEFENE Events	NALMEFENE Total	PLACEBO Events	PLACEBO Total	RR	95%−CI	W(fixed)	W(random)
Gual (12023A)	6	341	11	337	0.54	[0.20; 1.44]	32.5%	24.7%
Mann (12014A)	17	302	16	296	1.04	[0.54; 2.02]	47.4%	54.1%
Karhuvaara (CPH−101−801)	8	242	4	161	1.33	[0.41; 4.35]	14.1%	17.0%
CPH−101−0701	1	85	2	82	0.48	[0.04; 5.22]	6.0%	4.2%
**Fixed effect model**		**970**		**876**	**0.89**	**[0.55; 1.43]**	**100%**	**−−**
**Random effects model**					**0.89**	**[0.55; 1.46]**	**−−**	**100%**

Heterogeneity: I−squared=0%, tau−squared=0, p=0.5907

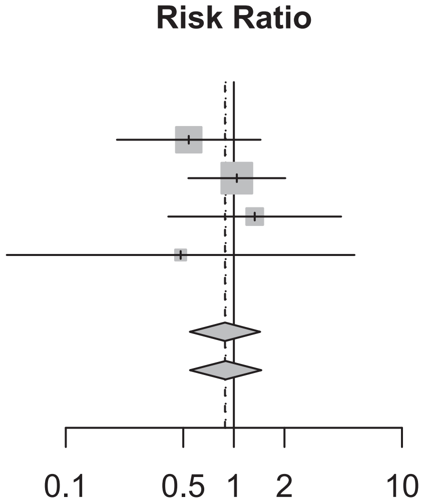

Serious adverse events (LFU=F) at 6 months

Study	NALMEFENE Events	NALMEFENE Total	PLACEBO Events	PLACEBO Total	RR	95%−CI	W(fixed)	W(random)
Gual (12023A)	23	358	34	360	0.68	[0.41; 1.13]	57.5%	51.9%
Mann (12014A)	21	306	18	298	1.14	[0.62; 2.09]	30.9%	36.2%
Karhuvaara (CPH−101−801)	8	242	4	161	1.33	[0.41; 4.35]	8.1%	9.6%
CPH−101−0701	1	85	2	82	0.48	[0.04; 5.22]	3.5%	2.4%
**Fixed effect model**		**991**		**901**	**0.87**	**[0.60; 1.25]**	**100%**	**−−**
**Random effects model**					**0.87**	**[0.60; 1.25]**	**−−**	**100%**

Heterogeneity: I−squared=0%, tau−squared=0, p=0.4997

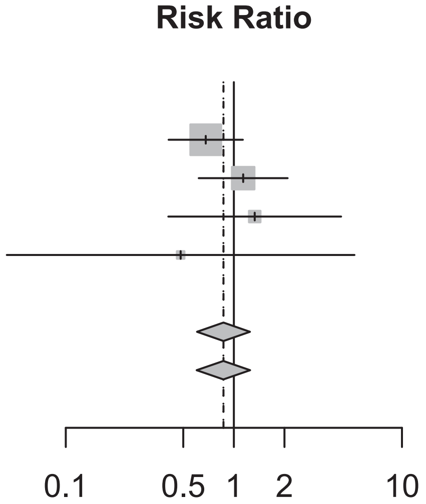

Withdrawal from the study at 6 months

Study	NALMEFENE Events	NALMEFENE Total	PLACEBO Events	PLACEBO Total	RR	95%−CI	W(fixed)	W(random)
Gual (12023A)	157	358	150	360	1.05	[0.89; 1.25]	41.8%	27.1%
Mann (12014A)	164	306	93	298	1.72	[1.41; 2.09]	26.3%	25.7%
Karhuvaara (CPH−101−801)	93	242	49	161	1.26	[0.95; 1.68]	16.5%	21.4%
CPH−101−0701	64	85	54	82	1.14	[0.94; 1.39]	15.4%	25.7%
**Fixed effect model**		**991**		**901**	**1.28**	**[1.15; 1.42]**	**100%**	**−−**
**Random effects model**					**1.27**	**[1.01; 1.59]**	**−−**	**100%**

Heterogeneity: I−squared=79.7%, tau−squared=0.0431, p=0.002

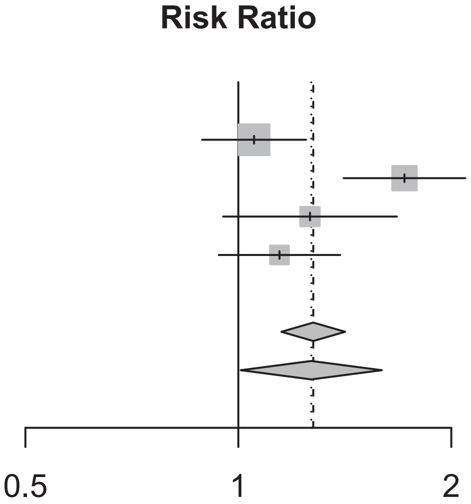

Withdrawal from the study for safety reasons at 6 months

Study	NALMEFENE Events	NALMEFENE Total	PLACEBO Events	PLACEBO Total	RR	95%−CI	W(fixed)	W(random)
Gual (12023A)	15	358	8	360	1.89	[0.81; 4.39]	22.9%	25.6%
Mann (12014A)	62	306	20	298	3.02	[1.87; 4.87]	58.1%	39.5%
Karhuvaara (CPH−101−801)	38	242	3	161	8.43	[2.65; 26.84]	10.3%	17.6%
CPH−101−0701	20	85	3	82	6.43	[1.99; 20.82]	8.8%	17.3%
**Fixed effect model**		**991**		**901**	**3.62**	**[2.50; 5.23]**	**100%**	**--**
**Random effects model**					**3.65**	**[2.02; 6.63]**	**--**	**100%**

Heterogeneity: I−squared=48.3%, tau−squared=0.1742, p=0.1218

Risk Ratio

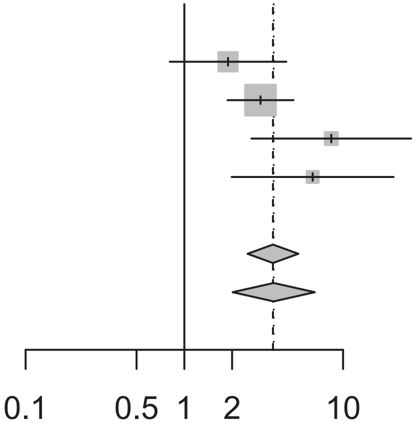

Mortality at 1 year

Study	NALMEFENE Events	NALMEFENE Total	PLACEBO Events	PLACEBO Total	RR	95%−CI
Van den Brink (12013A)	1	509	0	166	0.98	[0.04; 23.95]

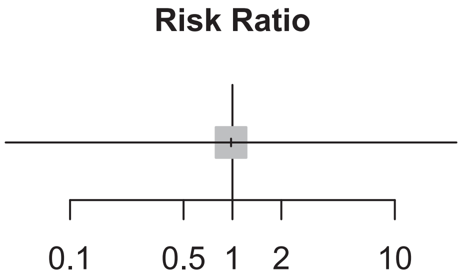

Monthly number of HDDs at 1 year

Study	NALMEFENE Total	NALMEFENE Mean	NALMEFENE SD	PLACEBO Total	PLACEBO Mean	PLACEBO SD	MD	95%−CI
Van den Brink (12013A)	265	−10.8	5.7	100	−9.2	5.3	−1.6	[−2.85; −0.35]

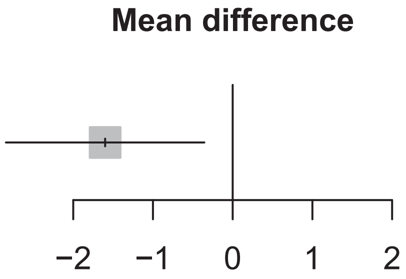

Total alcohol consumption at 1 year

Study	NALMEFENE Total	NALMEFENE Mean	NALMEFENE SD	PLACEBO Total	PLACEBO Mean	PLACEBO SD	SMD	95%−CI
Van den Brink (12013A)	265	−52.7	27.3	100	−49.1	26	−0.13	[−0.36; 0.1]

Standardised mean difference

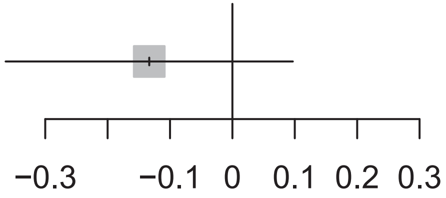

Response at 1 year

	NALMEFENE		PLACEBO		Risk Ratio		
Study	Events	Total	Events	Total		RR	95%−CI
Van den Brink (12013A)	345	415	104	137		1.1	[0.99; 1.21]

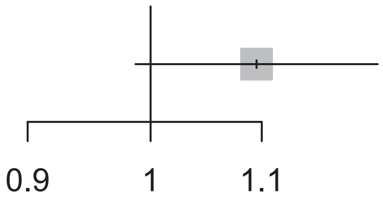

Clinical Global Impression−Severity score at 1 year

Study	NALMEFENE Total	NALMEFENE Mean	NALMEFENE SD	PLACEBO Total	PLACEBO Mean	PLACEBO SD	MD	95%−CI
Van den Brink (12013A)	258	−1.3	1	95	−1.1	1	−0.2	[−0.44; 0.04]

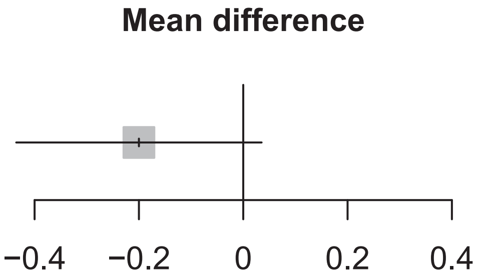

Carbohydrate−deficient transferrin at 1 year

	NALMEFENE			PLACEBO				
Study	Total	Mean	SD	Total	Mean	SD	SMD	95%−CI
Van den Brink (12013A)	255	0.15	1.4	94	0.41	1.4	−0.19	[−0.42; 0.05]

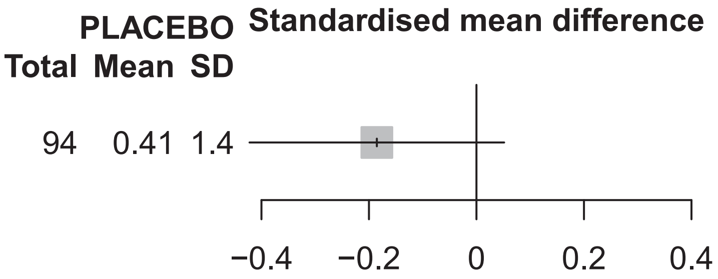

Adverse events at 1 year

Study	NALMEFENE Events	NALMEFENE Total	PLACEBO Events	PLACEBO Total	RR	95%−CI
Van den Brink (12013A)	377	501	103	164	1.2	[1.05; 1.36]

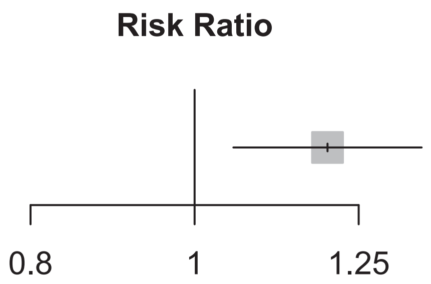

Adverse events (LFU=F) at 1 year

Study	NALMEFENE Events	NALMEFENE Total	PLACEBO Events	PLACEBO Total	RR	95%−CI
Van den Brink (12013A)	385	509	105	166	1.2	[1.05; 1.36]

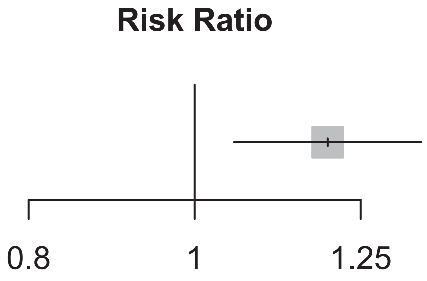

Serious adverse events at 1 year

	NALMEFENE		PLACEBO				
Study	Events	Total	Events	Total	Risk Ratio	RR	95%−CI
Van den Brink (12013A)	34	501	8	164		1.39	[0.66; 2.94]

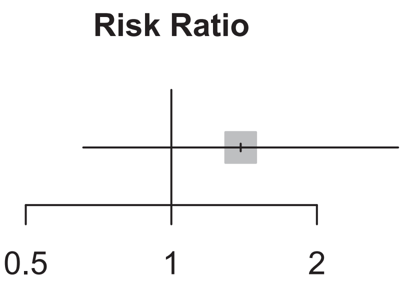

Serious adverse events (LFU=F) at 1 year

	NALMEFENE		PLACEBO			
Study	Events	Total	Events	Total	RR	95%−CI
Van den Brink (12013A)	42	509	10	166	1.37	[0.7; 2.67]

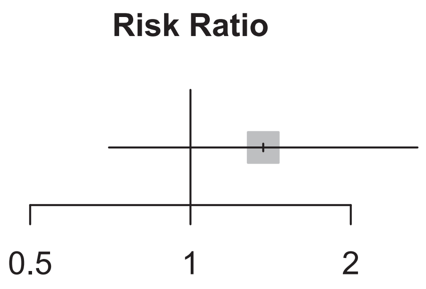

Withdrawal from the study at 1 year

Study	NALMEFENE Events	NALMEFENE Total	PLACEBO Events	PLACEBO Total	RR	95%−CI
Van den Brink (12013A)	199	509	54	166	1.2	[0.94; 1.53]

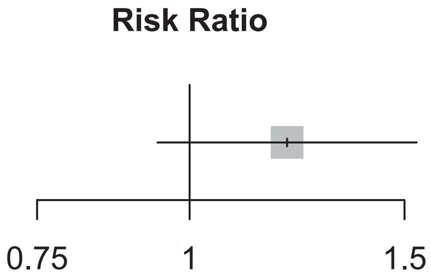

Withdrawal from the study for safety reasons at 1 year

	NALMEFENE		PLACEBO			
**Study**	**Events**	**Total**	**Events**	**Total**	**RR**	**95%−CI**
Van den Brink (12013A)	43	509	2	166	7.01	[1.72; 28.63]

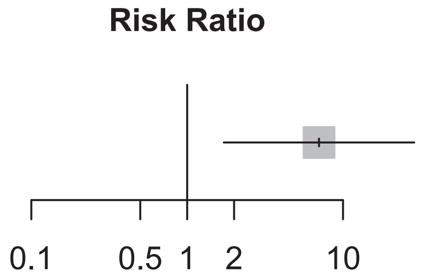

SENSITIVITY ANALYSIS

List of outcomes for which data involving studies lasting less than 6 months are available:

Health outcomes:

- Mortality ;

Alcohol consumption outcomes:

- Monthly number of HDDs ;
- Total alcohol consumption ;
- DrInC score ;
- Alcohol Dependence Scale score ;

Biological outcomes:

- γ-Glutamyltransferase ;
- Alanine-aminotransferase ;
- Mean corpuscular volume ;
- Carbohydrate-deficient transferrin ;

Treatment safety outcomes:

- Adverse events ;
- Serious adverse events ;
- Withdrawal from the study ;
- Withdrawal from the study for safety reasons.

Mortality

Study	NALMEFENE Events	NALMEFENE Total	PLACEBO Events	PLACEBO Total	RR	95%−CI	W(fixed)	W(random)
Gual (12023A)	1	358	1	360	1.01	[0.06; 16.01]	19.7%	38.6%
Mann (12014A)	0	306	2	298	0.19	[0.01; 4.04]	50.1%	32.2%
Karhuvaara (CPH−101−801)	0	242	0	161			0.0%	0.0%
Mason 1994	0	7	0	7			0.0%	0.0%
Mason 1999	0	70	0	35			0.0%	0.0%
Anton (CPH−101−0299)	0	66	0	68			0.0%	0.0%
CPH−101−0399	0	50	0	50			0.0%	0.0%
CPH−101−0701	0	85	1	82	0.32	[0.01; 7.78]	30.2%	29.2%
**Fixed effect model**		**1184**		**1061**	**0.39**	**[0.08; 2.01]**	**100%**	**−−**
**Random effects model**					**0.43**	**[0.08; 2.38]**	**−−**	**100%**

Heterogeneity: I−squared=0%, tau−squared=0, p=0.7176

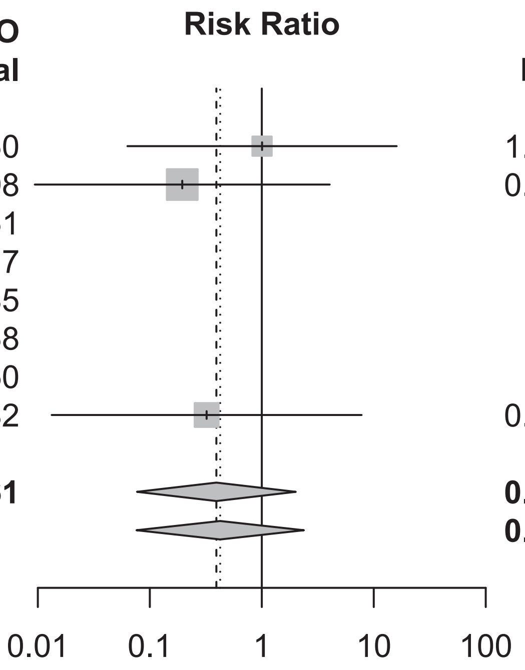

Monthly number of HDDs

Study	NALMEFENE Total	NALMEFENE Mean	NALMEFENE SD	PLACEBO Total	PLACEBO Mean	PLACEBO SD	MD	95%−CI	W(fixed)	W(random)
Gual (12023A)	212	−12.3	7.3	229	−10.6	7.6	−1.70	[−3.09; −0.31]	27.5%	27.5%
Mann (12014A)	152	−11.2	7.4	213	−8.9	8.8	−2.30	[−3.97; −0.63]	19.1%	19.1%
Van den Brink (12013A)	320	−9.8	7.2	110	−8.9	6.3	−0.90	[−2.32; 0.52]	26.5%	26.5%
Karhuvaara (CPH−101−801)	147	−6.1	7.7	114	−4.2	7.4	−1.90	[−3.74; −0.06]	15.7%	15.7%
Anton (CPH−101−0299)	51	−14.1	10.1	48	−13.4	10.8	−0.70	[−4.83; 3.43]	3.1%	3.1%
CPH−101−0399	46	−5.6	7.8	45	−3.6	7.6	−2.00	[−5.16; 1.16]	5.3%	5.3%
CPH−101−0701	30	−10.9	9.3	37	−8.5	9.1	−2.40	[−6.84; 2.04]	2.7%	2.7%
**Fixed effect model**	**958**			**796**			**−1.64**	**[−2.37; −0.91]**	**100%**	**--**
**Random effects model**							**−1.64**	**[−2.37; −0.91]**	**--**	**100%**

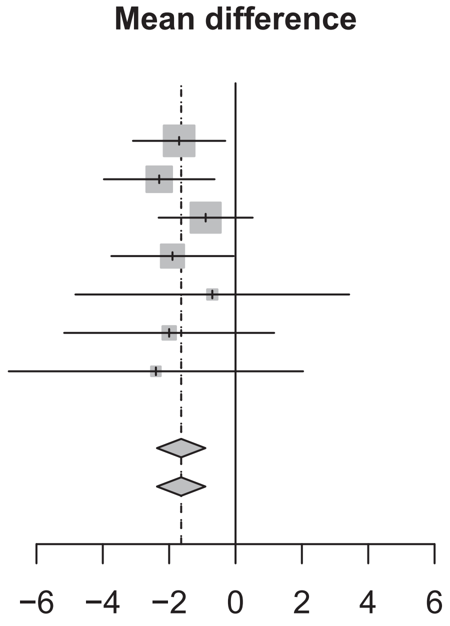

Heterogeneity: I−squared=0%, tau−squared=0, p=0.9108

Total alcohol consumption

Study	NALMEFENE Total	NALMEFENE Mean	NALMEFENE SD	PLACEBO Total	PLACEBO Mean	PLACEBO SD	SMD	95%−CI	W(fixed)	W(random)
Gual (12023A)	212	−59.0	33.5	229	−54.0	33.3	−0.15	[−0.34; 0.04]	27.0%	27.0%
Mann (12014A)	152	−50.7	29.6	213	−39.7	32.1	−0.35	[−0.56; −0.14]	21.5%	21.5%
Van den Brink (12013A)	320	−49.0	28.6	110	−45.6	27.3	−0.12	[−0.34; 0.10]	20.1%	20.1%
Karhuvaara (CPH−101−801)	149	−16.3	24.0	114	−11.3	22.5	−0.21	[−0.46; 0.03]	15.8%	15.8%
Anton (CPH−101−0299)	51	−40.0	36.0	48	−46.0	46.0	0.14	[−0.25; 0.54]	6.1%	6.1%
CPH−101−0399	46	−14.3	19.6	45	−9.4	25.7	−0.21	[−0.63; 0.20]	5.6%	5.6%
CPH−101−0701	30	−44.5	48.0	37	−41.6	49.0	−0.06	[−0.54; 0.42]	4.1%	4.1%
**Fixed effect model**	**960**			**796**			**−0.18**	**[−0.28; −0.08]**	**100%**	**−−**
**Random effects model**							**−0.18**	**[−0.28; −0.08]**	**−−**	**100%**

Heterogeneity: I−squared=0%, tau−squared=0, p=0.4285

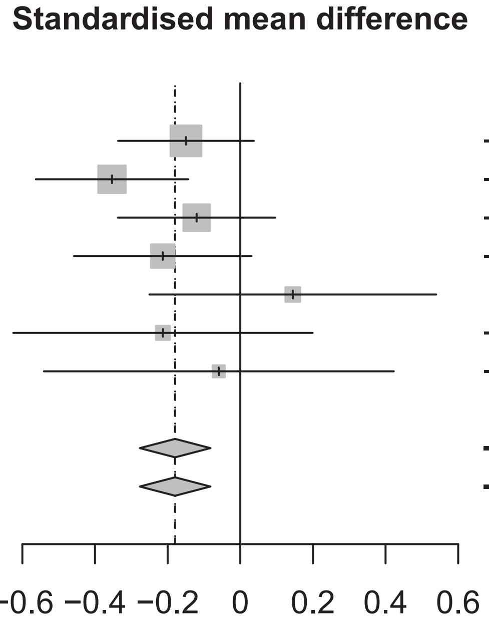

DrInC score

Study	NALMEFENE Total	NALMEFENE Mean	NALMEFENE SD	PLACEBO Total	PLACEBO Mean	PLACEBO SD	SMD	95%−CI	W(fixed)	W(random)
Gual (12023A)	209	−20.4	21.7	230	−19.0	22.7	−0.06	[−0.25; 0.12]	26.1%	26.1%
Mann (12014A)	158	−16.0	13.8	213	−13.8	14.6	−0.15	[−0.36; 0.05]	21.6%	21.6%
Van den Brink (12013A)	318	−18.8	23.2	108	−19.5	21.8	0.03	[−0.19; 0.25]	19.3%	19.3%
Karhuvaara (CPH−101−801)	161	24.7	17.4	115	30.2	20.1	−0.30	[−0.54; −0.05]	15.9%	15.9%
Anton (CPH−101−0299)	51	19.0	20.0	52	24.0	20.0	−0.25	[−0.64; 0.14]	6.1%	6.1%
CPH−101−0399	46	22.9	17.9	45	29.0	22.3	−0.30	[−0.71; 0.11]	5.4%	5.4%
CPH−101−0701	49	29.5	23.5	47	36.5	28.0	−0.27	[−0.67; 0.13]	5.7%	5.7%
**Fixed effect model**	**992**			**810**			**−0.14**	**[−0.23; −0.04]**	**100%**	**−−**
**Random effects model**							**−0.14**	**[−0.23; −0.04]**	**−−**	**100%**

Heterogeneity: I−squared=0%, tau−squared=0, p=0.4368

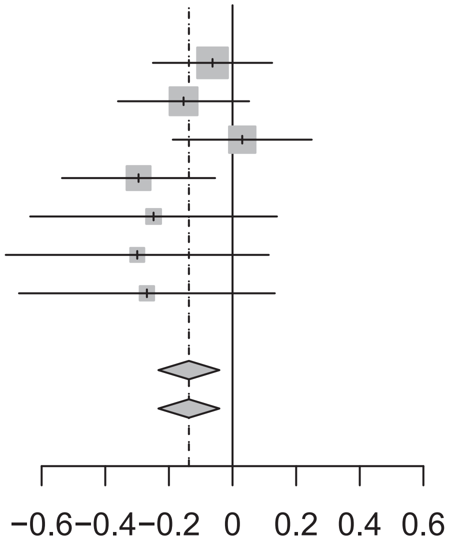

Alcohol Dependence Scale score

Study	NALMEFENE Total	NALMEFENE Mean	NALMEFENE SD	PLACEBO Total	PLACEBO Mean	PLACEBO SD	SMD	95%−CI	W(fixed)	W(random)
Gual (12023A)	328	−5.9	7.2	325	−5.1	7.2	−0.11	[−0.26; 0.04]	38.2%	38.2%
Mann (12014A)	290	−4.6	5.1	289	−4.3	5.1	−0.06	[−0.22; 0.10]	33.9%	33.9%
Karhuvaara (CPH−101−801)	167	11.6	6.7	120	13.1	7.0	−0.22	[−0.45; 0.02]	16.3%	16.3%
Anton (CPH−101−0299)	51	11.0	7.5	50	12.0	6.8	−0.14	[−0.53; 0.25]	5.9%	5.9%
CPH−101−0701	49	12.2	9.0	47	13.3	9.5	−0.12	[−0.52; 0.28]	5.6%	5.6%
**Fixed effect model**	**885**			**831**			**−0.11**	**[−0.21; −0.02]**	**100%**	**−−**
**Random effects model**							**−0.11**	**[−0.21; −0.02]**	**−−**	**100%**

Heterogeneity: I−squared=0%, tau−squared=0, p=0.8739

Standardised mean difference

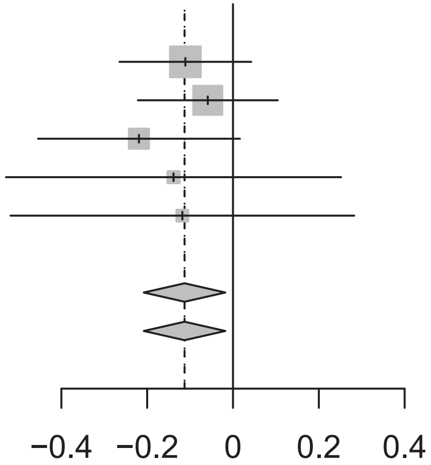

Gamma−Glutamyltransferase

Study	NALMEFENE Total	NALMEFENE Mean	NALMEFENE SD	PLACEBO Total	PLACEBO Mean	PLACEBO SD	MD	95%−CI	W(fixed)	W(random)
Gual (12023A)	207	43.4	NA	224	44.9	NA	−1.50		0.0%	0.0%
Mann (12014A)	158	40.3	NA	211	45.7	NA	−5.40		0.0%	0.0%
Van den Brink (12013A)	319	32.2	NA	108	34.5	NA	−2.30		0.0%	0.0%
Karhuvaara (CPH−101−801)	148	77.2	89.1	115	115.4	189.8	−38.20	[−75.74; −0.66]	25.8%	29.6%
Anton (CPH−101−0299)	47	44.4	46.3	50	58.4	64.0	−14.00	[−36.13; 8.13]	74.2%	70.4%
CPH−101−0399	45	55.0	NA	44	53.0	NA	2.00		0.0%	0.0%
**Fixed effect model**	**924**			**752**			**−20.24**	**[−39.31; −1.18]**	**100%**	**--**
**Random effects model**							**−21.15**	**[−42.80; 0.49]**	**--**	**100%**

Heterogeneity: I−squared=15.6%, tau−squared=45.61, p=0.2764

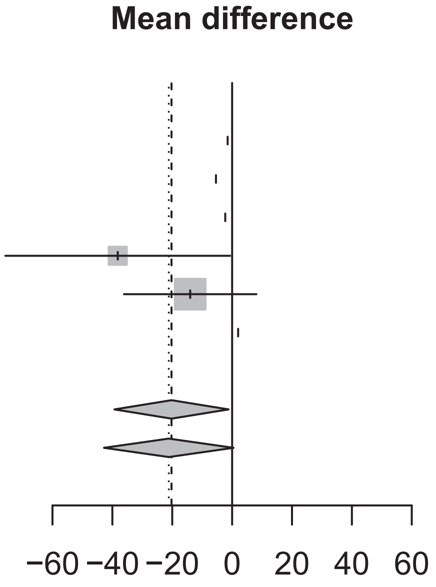

Alanine−aminotransferase

Study	NALMEFENE Total	NALMEFENE Mean	NALMEFENE SD	PLACEBO Total	PLACEBO Mean	PLACEBO SD	MD	95%−CI	W(fixed)	W(random)
Gual (12023A)	205	25.0	NA	222	27.2	NA	−2.20		0.0%	0.0%
Mann (12014A)	158	25.4	NA	209	28.1	NA	−2.70		0.0%	0.0%
Van den Brink (12013A)	318	25.6	NA	108	25.8	NA	−0.20		0.0%	0.0%
Karhuvaara (CPH−101−801)	148	37.5	24.5	115	52.0	45.4	−14.50	[−23.69; −5.31]	42.6%	48.1%
Anton (CPH−101−0299)	47	24.7	22.1	50	27.0	17.2	−2.30	[−10.22; 5.62]	57.4%	51.9%
CPH−101−0399	45	34.0	NA	44	27.5	NA	6.50		0.0%	0.0%
**Fixed effect model**	**921**			**748**			**−7.50**	**[−13.49; −1.50]**	**100%**	**−−**
**Random effects model**							**−8.17**	**[−20.11; 3.78]**	**−−**	**100%**

Heterogeneity: I−squared=74.3%, tau−squared=55.28, p=0.0486

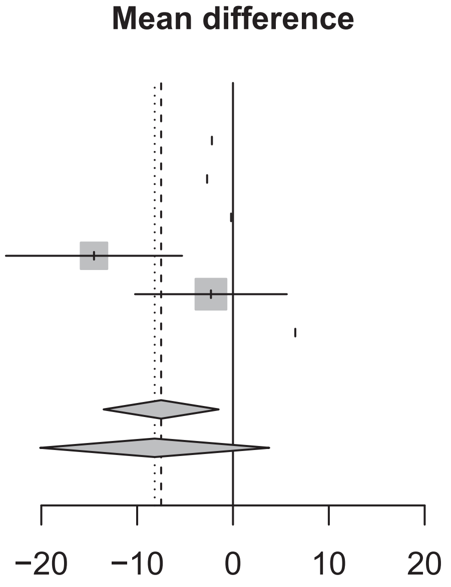

Mean corpuscular volume

Study	NALMEFENE Total	NALMEFENE Mean	NALMEFENE SD	PLACEBO Total	PLACEBO Mean	PLACEBO SD	MD	95%−CI	W(fixed)	W(random)
Gual (12023A)	193	97.2	NA	215	97.3	NA	−0.10		0.0%	0.0%
Mann (12014A)	145	96.5	NA	204	96.7	NA	−0.20		0.0%	0.0%
Van den Brink (12013A)	296	95.5	NA	103	95.6	NA	−0.10		0.0%	0.0%
Karhuvaara (CPH−101−801)	147	96.1	3.8	114	96.3	4.9	−0.20	[−1.29; 0.89]	77.5%	77.5%
Anton (CPH−101−0299)	49	96.0	5.0	49	96.0	5.2	0.00	[−2.02; 2.02]	22.5%	22.5%
CPH−101−0399	45	95.0	NA	44	96.5	NA	−1.50		0.0%	0.0%
**Fixed effect model**	**875**			**729**			**−0.15**	**[−1.11; 0.80]**	**100%**	**−−**
**Random effects model**							**−0.15**	**[−1.11; 0.80]**	**−−**	**100%**

Heterogeneity: I−squared=0%, tau−squared=0, p=0.8644

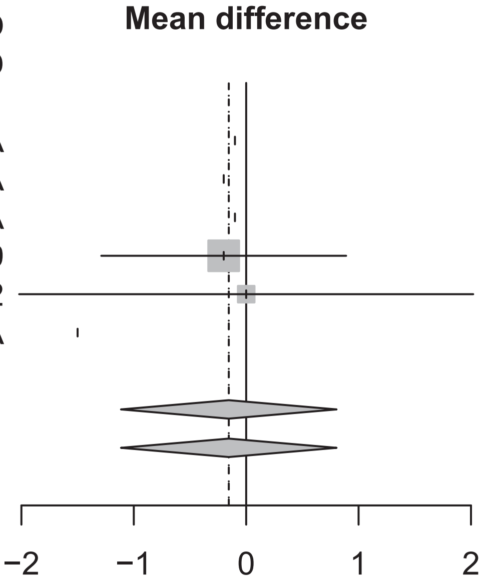

Carbohydrate−deficient transferrin

Study	NALMEFENE Total	NALMEFENE Mean	NALMEFENE SD	PLACEBO Total	PLACEBO Mean	PLACEBO SD	SMD	95%−CI	W(fixed)	W(random)
Gual (12023A)	195	0.10	1.4	216	0.00	1.5	0.07	[−0.12; 0.26]	28.7%	24.7%
Mann (12014A)	152	0.00	1.2	207	0.10	1.4	−0.08	[−0.29; 0.13]	24.6%	23.1%
Van den Brink (12013A)	313	−0.14	1.2	106	0.13	1.1	−0.23	[−0.45; −0.01]	22.1%	22.0%
Karhuvaara (CPH−101−801)	148	3.10	1.8	115	3.30	2.0	−0.11	[−0.35; 0.14]	18.1%	19.9%
Anton (CPH−101−0299)	47	3.20	1.8	47	2.70	0.9	0.35	[−0.06; 0.76]	6.5%	10.3%
CPH−101−0399	45	18.00	NA	44	20.00	NA			0.0%	0.0%
**Fixed effect model**	**900**			**735**			**−0.05**	**[−0.15; 0.06]**	**100%**	**−−**
**Random effects model**							**−0.04**	**[−0.19; 0.11]**	**−−**	**100%**

Heterogeneity: I−squared=49.4%, tau−squared=0.0142, p=0.0954

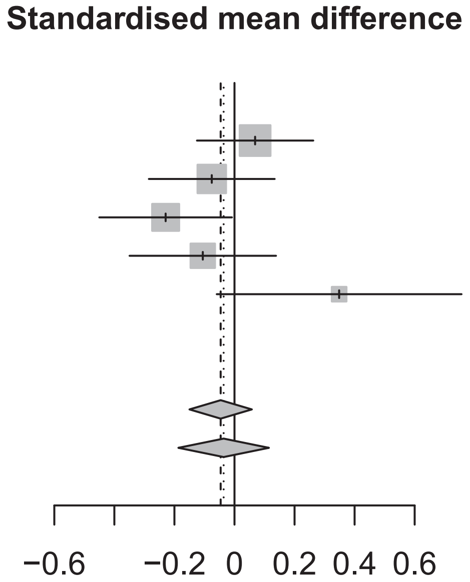

Adverse events

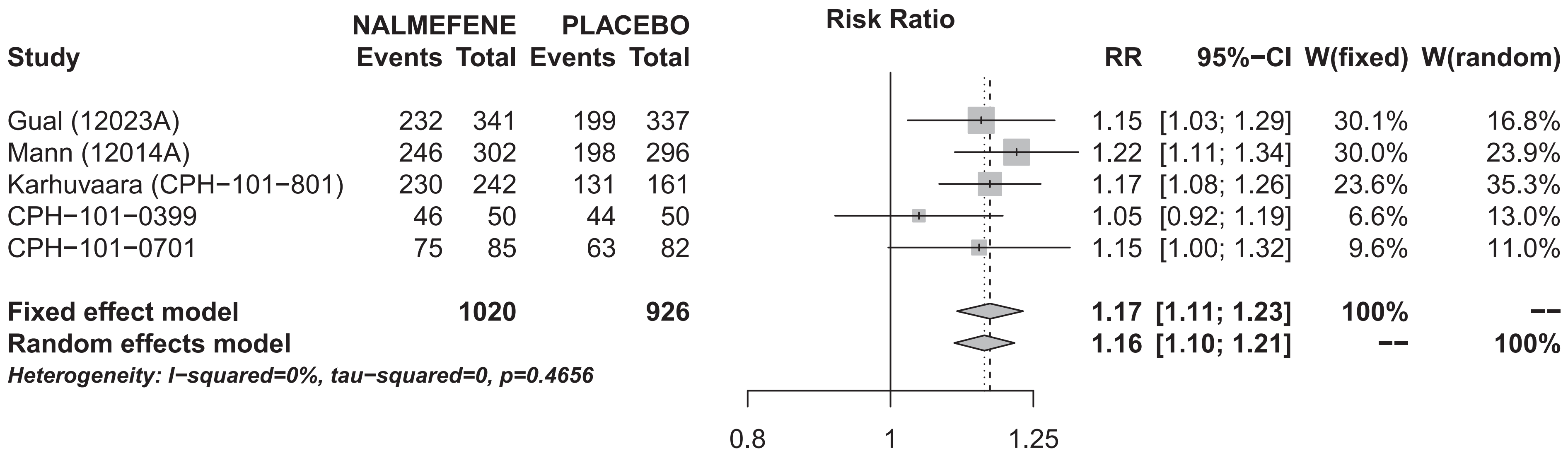

Adverse events (LFU=F)

Study	NALMEFENE Events	NALMEFENE Total	PLACEBO Events	PLACEBO Total	RR	95%−CI	W(fixed)	W(random)
Gual (12023A)	249	358	222	360	1.13	[1.01; 1.25]	32.1%	19.0%
Mann (12014A)	250	306	200	298	1.22	[1.11; 1.34]	29.4%	23.6%
Karhuvaara (CPH−101−801)	230	242	131	161	1.17	[1.08; 1.26]	22.8%	34.2%
CPH−101−0399	46	50	44	50	1.05	[0.92; 1.19]	6.4%	12.5%
CPH−101−0701	75	85	63	82	1.15	[1.00; 1.32]	9.3%	10.7%
**Fixed effect model**		**1041**		**951**	**1.16**	**[1.10; 1.22]**	**100%**	**−−**
**Random effects model**					**1.15**	**[1.10; 1.21]**	**−−**	**100%**

Heterogeneity: I−squared=0%, tau−squared=0, p=0.446

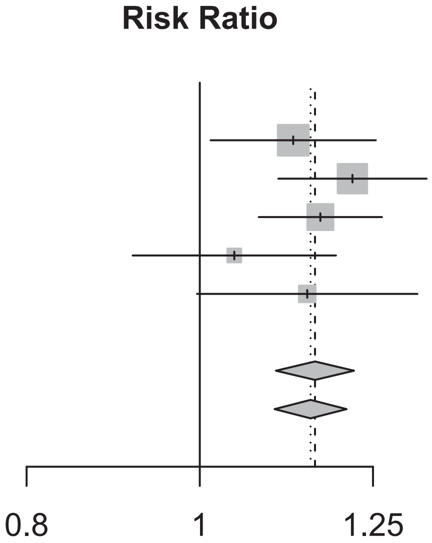

Serious adverse events

Study	NALMEFENE Events	NALMEFENE Total	PLACEBO Events	PLACEBO Total	RR	95%−CI	W(fixed)	W(random)
Gual (12023A)	6	341	11	337	0.54	[0.20; 1.44]	27.6%	23.4%
Mann (12014A)	17	302	16	296	1.04	[0.54; 2.02]	40.3%	51.4%
Karhuvaara (CPH−101−801)	8	242	4	161	1.33	[0.41; 4.35]	12.0%	16.2%
Mason 1999	0	70	0	35			0.0%	0.0%
Anton (CPH−101−0299)	1	66	0	68	3.09	[0.13; 74.52]	1.2%	2.2%
CPH−101−0399	0	50	5	50	0.09	[0.01; 1.60]	13.7%	2.8%
CPH−101−0701	1	85	2	82	0.48	[0.04; 5.22]	5.1%	4.0%
**Fixed effect model**		**1156**		**1029**	**0.80**	**[0.51; 1.27]**	**100%**	**−−**
**Random effects model**					**0.86**	**[0.54; 1.39]**	**−−**	**100%**

Heterogeneity: I−squared=0%, tau−squared=0, p=0.4161

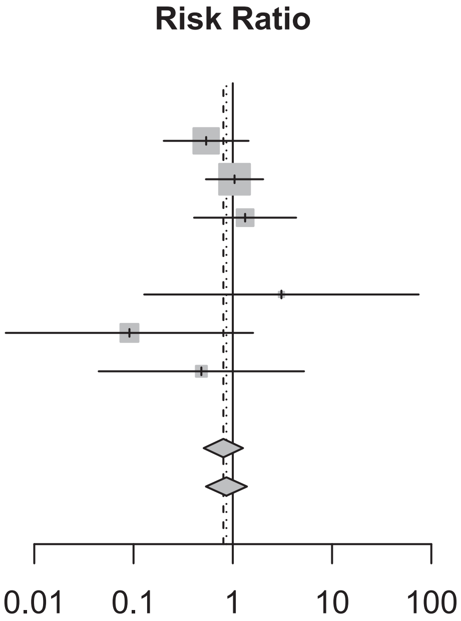

Serious adverse events (LFU=F)

Study	NALMEFENE Events	NALMEFENE Total	PLACEBO Events	PLACEBO Total	RR	95%−CI	W(fixed)	W(random)
Gual (12023A)	23	358	34	360	0.68	[0.41; 1.13]	52.2%	47.3%
Mann (12014A)	21	306	18	298	1.14	[0.62; 2.09]	28.1%	35.6%
Karhuvaara (CPH−101−801)	8	242	4	161	1.33	[0.41; 4.35]	7.4%	10.8%
Mason 1999	0	70	0	35			0.0%	0.0%
Anton (CPH−101−0299)	1	66	0	68	3.09	[0.13; 74.52]	0.8%	1.6%
CPH−101−0399	0	50	5	50	0.09	[0.01; 1.60]	8.5%	1.9%
CPH−101−0701	1	85	2	82	0.48	[0.04; 5.22]	3.1%	2.8%
**Fixed effect model**		**1177**		**1054**	**0.82**	**[0.58; 1.16]**	**100%**	**−−**
**Random effects model**					**0.86**	**[0.57; 1.28]**	**−−**	**100%**

Heterogeneity: I−squared=7.1%, tau−squared=0.0212, p=0.3709

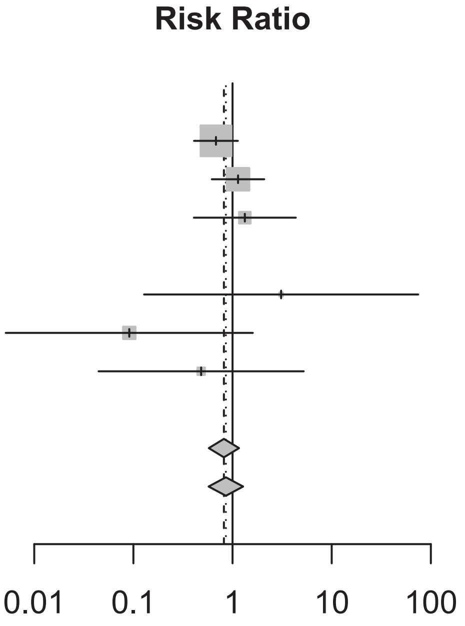

Withdrawal from the study

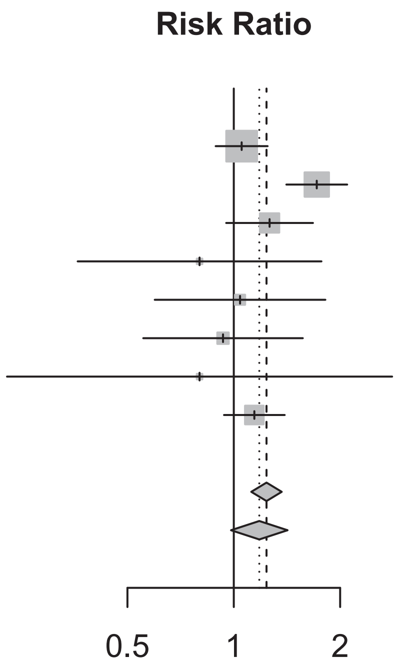

Study	NALMEFENE Events	NALMEFENE Total	PLACEBO Events	PLACEBO Total	RR	95%−CI	W(fixed)	W(random)
Gual (12023A)	157	358	150	360	1.05	[0.89; 1.25]	37.0%	21.3%
Mann (12014A)	164	306	93	298	1.72	[1.41; 2.09]	23.3%	20.0%
Karhuvaara (CPH−101−801)	93	242	49	161	1.26	[0.95; 1.68]	14.6%	16.1%
Mason 1994	4	7	5	7	0.80	[0.36; 1.77]	1.2%	4.5%
Mason 1999	25	70	12	35	1.04	[0.60; 1.82]	4.0%	7.7%
Anton (CPH−101−0299)	19	66	21	68	0.93	[0.55; 1.57]	5.1%	8.5%
CPH−101−0399	4	50	5	50	0.80	[0.23; 2.81]	1.2%	2.0%
CPH−101−0701	64	85	54	82	1.14	[0.94; 1.39]	13.6%	20.0%
**Fixed effect model**		**1184**		**1061**	**1.24**	**[1.12; 1.37]**	**100%**	**−−**
**Random effects model**					**1.18**	**[0.98; 1.42]**	**−−**	**100%**

Heterogeneity: I−squared=60.6%, tau−squared=0.034, p=0.013

Withdrawal from the study for safety reasons

Study	NALMEFENE Events	NALMEFENE Total	PLACEBO Events	PLACEBO Total	RR	95%−CI	W(fixed)	W(random)
Gual (12023A)	15	358	8	360	1.89	[0.81; 4.39]	19.2%	17.5%
Mann (12014A)	62	306	20	298	3.02	[1.87; 4.87]	48.8%	46.5%
Karhuvaara (CPH−101−801)	38	242	3	161	8.43	[2.65; 26.84]	8.7%	9.7%
Mason 1994	1	7	1	7	1.00	[0.08; 13.02]	2.4%	2.0%
Mason 1999	3	70	0	35	3.52	[0.19; 66.38]	1.6%	1.6%
Anton (CPH−101−0299)	9	66	2	68	4.64	[1.04; 20.66]	4.7%	5.9%
CPH−101−0399	6	50	3	50	2.00	[0.53; 7.56]	7.2%	7.4%
CPH−101−0701	20	85	3	82	6.43	[1.99; 20.82]	7.4%	9.4%
**Fixed effect model**		**1184**		**1061**	**3.48**	**[2.48; 4.89]**	**100%**	**−−**
**Random effects model**					**3.21**	**[2.22; 4.65]**	**−−**	**100%**

Heterogeneity: I−squared=5%, tau−squared=0.0164, p=0.3915

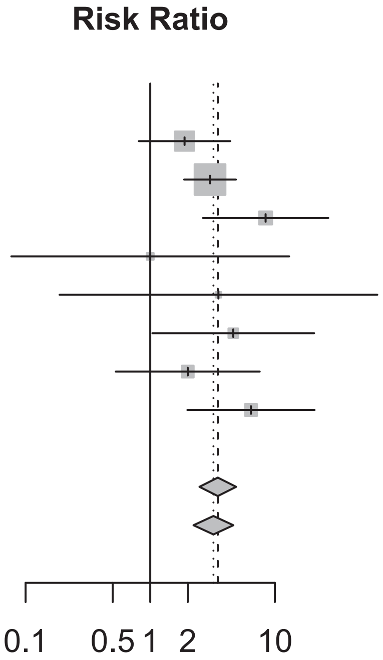

Supplement: S1 Supporting Information — Includes further information regarding the search strategy and results from the main and sensitivity analyses. (PDF) [file pmed.1001924.s001.pdf]
