# Supplementary material for: Risks and Benefits of Nalmefene in the Treatment of Adult Alcohol Dependence: A Systematic Literature Review and Meta-Analysis of Published and Unpublished Double-Blind Randomized Controlled Trials
Source: PLoS Med. 2015 Dec 22;12(12):e1001924. doi: 10.1371/journal.pmed.1001924 (PMC4687857; doi:10.1371/journal.pmed.1001924)
Supplement: S2 Supporting Information — (DOC) [file pmed.1001924.s002.doc]

***Dose-response analysis: network geometry.***

***The network is closed, with placebo as a central node. Estimations of differences between as-needed 20 mg nalmefene and other administration regimens are based on indirect comparisons.***

***Dose-response analysis:*** *Forest plot for heavy drinking days with nalmefene 20 mg as-needed as the reference*

***Results of the network meta-analysis (fixed effect model) for heavy drinking days (HDD) are the Standardised Mean Differences (SMD) with their 95% confidence interval (CI).***

***I²= 0 % p-value (Q) = 0.83.***
