# Supplementary material for: Risks and Benefits of Nalmefene in the Treatment of Adult Alcohol Dependence: A Systematic Literature Review and Meta-Analysis of Published and Unpublished Double-Blind Randomized Controlled Trials
Source: PLoS Med. 2015 Dec 22;12(12):e1001924. doi: 10.1371/journal.pmed.1001924 (PMC4687857; doi:10.1371/journal.pmed.1001924)
Supplement: S1 Text — (PDF) [file pmed.1001924.s003.pdf]

Nalmefene in the treatment of adults alcohol dependence in order to reduce alcohol consumption: a systematic review and meta-analysis

Florian Naudet, Clement Palpacuer, Remy Boussageon

Citation

Florian Naudet, Clement Palpacuer, Remy Boussageon. Nalmefene in the treatment of adults alcohol dependence in order to reduce alcohol consumption: a systematic review and meta-analysis. PROSPERO 2014:CRD42014014853 Available from http://www.crd.york.ac.uk/PROSPERO_REBRANDING/display_record.asp?ID=CRD42014014853

Review question(s)

Nalmefene has recently been approved by the EMA in the treatment of alcohol dependence in order to help reduce alcohol consumption in adults with alcohol dependence who consumes more than 60 g of alcohol per day (for men) or more than 40 g per day (for women). It should only be used together with psychosocial support (counselling) and only in people who do not have physical withdrawal symptoms and who do not require immediate detoxification. It is the first approved treatment which could prevent binge drinking by reducing the number of drinks within one session. A reduction in HDD is supposed to have a huge health benefit in patients with alcohol dependence, including an impact on personal health. On the other hand, the EMA approval was polemic since the average benefit from nalmefene was modest, as for other drugs in this field. The subject thus deserves a meta-analysis using clinically relevant outcomes to explore its interest in term of public health.

Searches

Databases:

- PubMed/MEDLINE, EMBASE, Cochrane Library.

- Search strategy/search terms as follows: 'Nalmefene (limitation: clinical trial)'

Unpublished studies:

- EMA, FDA

- Industrial (Lundbeck), authors

Types of study to be included

Prospective double blind randomised controlled trials against placebo

Condition or domain being studied

Nalmefene for alcohol dependance

Participants/ population

Patients with alcohol dependence (non abstinent)

Intervention(s), exposure(s)

Oral nalmefene

Comparator(s)/ control

Placebo

Active comparator if present

Context

All patients with alcohol dependence (non abstinent)

Outcome(s)

Primary outcomes

1/ Health outcomes

- Accidents (including motor vehicle crashes) or injuries

- QoL or function (SF-36)

- Mortality

- Somatic alcoholism' complications

- 6 monthes

- 1 year (when available)

- More than one year (when available)

Secondary outcomes

2/ Intermediate biological outcomes

- Gamma-glutamyltransferase (GGT)

- ALAT

- Mean Corpuscular Volume (MCV)

- Carbohydrate-deficient transferrin CDT

3/ Intermediate clinical outcomes: alcohol consumption

– Monthly number of heavy drinking days (HDD), defined as a day with alcohol consumption of 60 g or more for males and 40 g or more for females.

– Total alcohol consumption (TAC), defined as mean daily alcohol consumption in grams/day over a month.

- The proportion of patients decreasing their consumption to low risk levels or no consumption

- Complete abstinence

4/ Alcohol dependence symptoms and clinical status

- Total Drinker Inventory of Consequences (DrInc) score

- Clinical Global Impression-Severity (CGI-S)

- Change from baseline in Alcohol Dependence Scale (ADS)

5/ Safety

- Treatment-emergent adverse events

- Serious adverse events

- Withdrawal

- Withdrawal for safety purpose

- 6 monthes

- 1 year (when available)

- More than one year (when available)

Data extraction, (selection and coding)

Selection and coding will be performed by two independent reviewers in a blinded manner. Disagreement will be resolved by consensus or in consultation with a third reviewer. Studies appearing to duplicate authors, treatment comparisons, sample sizes and outcomes will be checked one against another to avoid double-counting and integrating data from several reports on the same study. A data extraction sheet based on the Cochrane Handbook for Systematic Reviews of Interventions guidelines will be developed. In case of missing data, the sponsor of the study and/or corresponding authors will be contacted.

For each included study, information will be extracted on:

1) characteristics of the study (year, country, Type of comparator(s), Number of arms, Funding);

2) characteristics of trial participants (age, gender, number of patients included in analysis, population of analysis);

3) type of intervention (treatment, duration);

4) outcomes measure as stated above.

Risk of bias (quality) assessment

Quality of the study will be assessed using an appropriate standardized critical appraisal instrument from the Cochrane Collaboration's ("The Cochrane Collaboration's tool for assessing risk of bias").

Strategy for data synthesis

Aggregated data will be used and a quantitative synthesis is planned.

The efficiency index used for our qualitative judgement criteria will be the Odds Ratio.

The efficiency index used for our quantitative judgement criteria will be the difference in means or standardised differences in means.

The estimate of overall effect (summary measure) will be made using fixed effect models (Mantel-Haenszel) and random effects (DerSimonian and Laird). The choice between the two models based on the presence (or absence) of heterogeneity diagnosed using: - a visual inspection of forest plots; - the Cochran Q test; - the I-squared index.

The analytic strategy presented in the paper will be considered as the gold standard in our analysis. As a substantial number of lost to follow up are expected, sensitivity analyses will be performed using more conservative approaches (BOCF for quantitative outcomes and lost to follow up = failure for qualitative outcomes).

Analysis of subgroups or subsets

None

Dissemination plans

Paper in a medical journal

Contact details for further information

Dr Naudet

Centre d'Investigation Clinique CIC-P INSERM 1414, Hôpital de Pontchaillou, Centre Hospitalier Universitaire de Rennes & Universit de Rennes 1

2 Rue Henri le Guilloux 35000 Rennes

Rennes, France

floriannaudet@gmail.com

Organisational affiliation of the review
INSERM

Review team
Dr Florian Naudet, CIC1414
Mr Clement Palpacuer, CIC1414
Dr Remy Boussageon, Poitiers University

Collaborators
Professor Bruno Laviolle, CIC1414
Dr Jean Michel Reymann, CIC1414

Details of any existing review of the same topic by the same authors
None

Anticipated or actual start date
10 November 2014

Anticipated completion date
12 May 2015

Funding sources/sponsors
None

Conflicts of interest
There are no conflicts of interest regarding this review. All authors have completed the Unified Competing Interest form at http://www.icmje.org/coi_disclosure.pdf (available on request from the corresponding author) and declare that (1) No authors have support from any company for the submitted work; (2) N.F. has relationships (board membership or Travel/accommodations expenses covered/reimbursed) with Servier, BMS, Lundbeck and Janssen who might have an interest in the work submitted in the previous 3 years ; B.R. and P.C. have no relationships with any company that might have an interest in the submitted work in the previous 3 years; (3) none of the authors' spouses, partners, or children have any financial relationships that may be relevant to the submitted work; and (4) none of the authors has any non-financial interests that may be relevant to the submitted work.

Language
English

Country
France

Subject index terms status
Subject indexing assigned by CRD

Subject index terms
Adult; Alcohol Drinking; Alcoholism; Humans; Naltrexone

Stage of review
Ongoing

Date of registration in PROSPERO
12 November 2014

Date of publication of this revision
12 November 2014

Stage of review at time of this submission	Started	Completed
Preliminary searches	No	No
Piloting of the study selection process	No	No
Formal screening of search results against eligibility criteria	No	No
Data extraction	No	No
Risk of bias (quality) assessment	No	No
Data analysis	No	No

PROSPERO
International prospective register of systematic reviews
The information in this record has been provided by the named contact for this review. CRD has accepted this information in good faith and registered the review in PROSPERO. CRD bears no responsibility or liability for the content of this registration record, any associated files or external websites.
